# Ranking land degradation drivers in eastern Inner Mongolia using partial order theory and Hasse diagram analysis

**DOI:** 10.1038/s41598-026-47280-5

**Published:** 2026-04-10

**Authors:** Kaixin Liu, Batunacun, Yong Mei, Yu Feng, Ruifang Guo, Chang An, Yaxin Wang, Bin Sun

**Affiliations:** 1 College of Geographical Science, Inner Mongolia Normal University, Hohhot, 010022 China; 2 State Key Laboratory of Resources Environmental, Institute of Geographic Sciences and Natural Resources Research, Chinese Academy of Sciences, Beijing, 100101 China; 3 Institute of Forest Resource Information Techniques, Chinese Academy of Forestry, Beijing, 100091 China; 4 Key Laboratory of Forestry Remote Sensing and Information System, NFGA, Beijing, 100091 China

**Keywords:** Land degradation, Driving factors, Partial order theory, Hasse diagram, Inner Mongolia, Ecology, Ecology, Environmental sciences, Environmental social sciences, Environmental studies, Geography, Geography

## Abstract

Land degradation (LD) poses a major challenge to global sustainable development, with the attainment of land degradation neutrality recognised as a key indicator of Sustainable Development Goal 15.3 (SDG 15.3). This goal focuses on combating desertification, restoring degraded land and soil, and including land affected by desertification, drought, and floods, with the aim of creating a land degradation-neutral world by 2030. Eastern Inner Mongolia (EIM), a typical agro-pastoral transitional zone, has experienced increasingly severe LD in recent decades. Identifying its dominant drivers is essential for improving ecological governance and land use management capacity. This study employed the Global 30 m Land-Cover Dynamics Monitoring Product (GLC_FCS30D) to characterise the spatio-temporal patterns of LD across three periods: 1990–2000, 2000–2010, and 2010–2020. Twelve drivers were identified and categorised into four groups: natural, human activities, economic, and urbanisation. Using Partial Order Theory and the Hasse Diagram Technique, the influence intensity of each driver group was systematically evaluated and ranked for each period. The combination of POT and the HDT enables transparent, interpretable, and reproducible ranking of multiple drivers while accounting for nonlinear, hierarchical, and interdependent relationships, offering advantages over conventional statistical or machine learning methods. The results showed that: (1) from 1990 to 2020, approximately 23% of EIM experienced land cover change, with degradation rates of 0.78%, 0.36%, and 0.41% for the three periods, respectively, and restoration rates of 0.71%, 0.36%, and 0.29%, respectively, the net land degradation and restoration areas were 5.83 × 10^4^ km^2^ and 4.62 × 10^4^ km^2^, respectively; (2) the dominant drivers of LD were ranked and spatially distributed differently across each league and city; (3) In the first two periods, the order of influence was: urbanisation > natural > human activities > economic. In the third period, the order changed to: urbanisation > natural > economic > human activities. This study reveals the spatiotemporal dynamics and dominant drivers of LD in EIM, providing a scientific basis for formulating regional land management and ecological restoration policies. It also offers valuable references for optimizing land use strategies, curbing LD, and achieving the sustainable development goals.

**Supplementary Information:**

The online version contains supplementary material available at 10.1038/s41598-026-47280-5.

## Introduction

Land degradation (LD) is recognised as one of the most severe socio-economic and environmental challenges worldwide^1^. Over the past three decades, approximately 30% of global land has been degraded, including 33% of grasslands, 25% of croplands, and 23% of forests^2^. This widespread degradation has profoundly affected human livelihoods, impacting nearly 1.5 billion people and causing estimated annual economic losses of around USD 300 billion^3^. LD also constitutes a major threat to human well-being and global food security^2^. For LD, conducting large-scale monitoring and identifying the underlying driving mechanisms are critical tasks^4^. However, because both LD and its drivers exhibit significant spatial and temporal complexities, accurately identifying these drivers across different spatial and temporal scales is essential for preventing further degradation and promoting land restoration and improvement^5^.

LD is recognised as a dynamic process characterised by a progressive decline in land productivity, driven by both environmental change and human activities^4^. It is a complex phenomenon arising from interactions between natural ecosystems and socio–economic systems, and is commonly defined as any reduction in soil quality, biological productivity, species diversity, human livelihoods, or the provision of ecosystem goods and services, with severity ranging from slight degradation to complete destruction or conversion to other land uses^6^. In this study, LD and land restoration (LR) are redefined based on the characteristics of land cover change in EIM. LD refers to the conversion of grassland, forest, and water bodies into cropland, bare areas, and impervious surfaces^5^. LR refers to the conversion of cropland and bare areas into grassland, forest, or water bodies^6^.

In LD research, the choice of scale plays a decisive role in identifying driving factors. At the macro scale (e.g. national or provincial levels), analyses highlight broad drivers such as climate change, policy orientation, and socio-economic development, but they often mask local variation^7^. At the micro scale (e.g. patch or pixel levels), studies can capture fine-grained processes such as household land management and land-use change, but their spatial coverage is limited^8^. Consequently, the influence of drivers differs across scales, and selecting an inappropriate scale may lead to misinterpretation of the underlying processes^8^. In this study, we emphasise the county (banner) scale as an intermediate level between macro and micro scales: it provides accessible statistical data, reflects regional heterogeneity, and corresponds to a key administrative unit for land management and policy implementation in China.

Moreover, the complexity of LD drivers manifests in five aspects: dynamic, integrality, hierarchy, synergy and spatial heterogeneity^5^. The dynamic aspect reflects variation in drivers across time and space, influenced by socio-economic conditions, climate change and human actions^9^. Integrality means that LD rarely results from a single cause; rather, it emerges from the combined influence of environmental, socio-economic, policy and other anthropogenic factors^10^. Hierarchy denotes that relationships among drivers are structured rather than random; each major driver group can be divided into sub-factors, forming a multi-level framework^11^. Synergy refers to interactive and mutually reinforcing effects among drivers that collectively contribute to LD^12^. Spatial heterogeneity refers to the uneven spatial distribution of drivers across different regions, ecosystems, and environmental conditions^13^. On this basis, we examine relationships between LD and twelve drivers, categorised into four groups: natural, human activities, economic and urbanisation.

Currently, a variety of methods are used to identify the driving factors of LD, including statistical approaches (e.g. linear regression, principal component analysis, and logistic regression), machine learning algorithms and the Geographical Detector method. Statistical approaches rely on linearity assumptions and often fail to capture the complex relationships between LD and its drivers. They also have limitations in representing regional economic factors^14^. Machine learning can handle large-scale, complex datasets^15^. However, model complexity and constraints in training data can lead to overfitting, whereby a model performs well on the training set but generalises poorly to unseen data^16^. The Geographical Detector is effective for assessing the influence of single factors and their pairwise interactions, but it may oversimplify the LD process when multi-factor interactions are involved^17^.

To address these limitations, this study employs Partial Order Theory (POT) and the Hasse Diagram Technique (HDT). POT ranks multiple indicators while considering nonlinear, hierarchical, and interdependent relationships among drivers^18^. HDT provides a visual representation of these relationships, allowing clear identification of the dominant drivers and their relative influence at the county level^19^. Unlike regression or machine learning models, which rely on linear assumptions or large training datasets, POT-HDT can capture nonlinear effects, account for the dynamic characteristics of drivers, and reveal the dominance relationships among driver groups using only a small amount of data^5,20^. In addition, unlike the Geographical Detector, which can only evaluate single factors or pairwise interactions, POT-HDT can rank multiple drivers simultaneously. These advantages make POT-HDT particularly suitable for identifying key LD drivers at the administrative scale and for supporting sustainable land management policies.

EIM is an ecologically fragile region, its inherent vulnerability, combined with climate change, making it highly prone to LD^21^. The extensive distribution of cropland and barren land has exacerbated wind erosion and desertification, highlighting the urgent need for effective regional land management^22^. Previous studies have focused on grassland net primary productivity, vegetation greenness trends, lignite resource development, and the spatio-temporal patterns of land reclamation and abandonment in EIM^23,24^. However, systematic identification of the dominant drivers of LD and their temporal dynamics in this region remains limited. In this study, we employ POT and HDT to identify the dominant driving factors of LD in the region. The specific objectives are: (1) to analyse the spatio-temporal dynamics of LD and its drivers; (2) to rank all drivers across different periods using partial order theory; and (3) to identify the dominant drivers of LD between 1990 and 2020. Based on regional policy changes and socio-economic development in EIM, we hypothesise that the relative influence of LD drivers has shifted over the study period. Specifically, we expect that while natural factors dominated LD in the early period (1990–2000), human-related drivers, particularly urbanisation and economic activities, have become increasingly dominant in the later period (2010–2020).

**Fig. 1:**
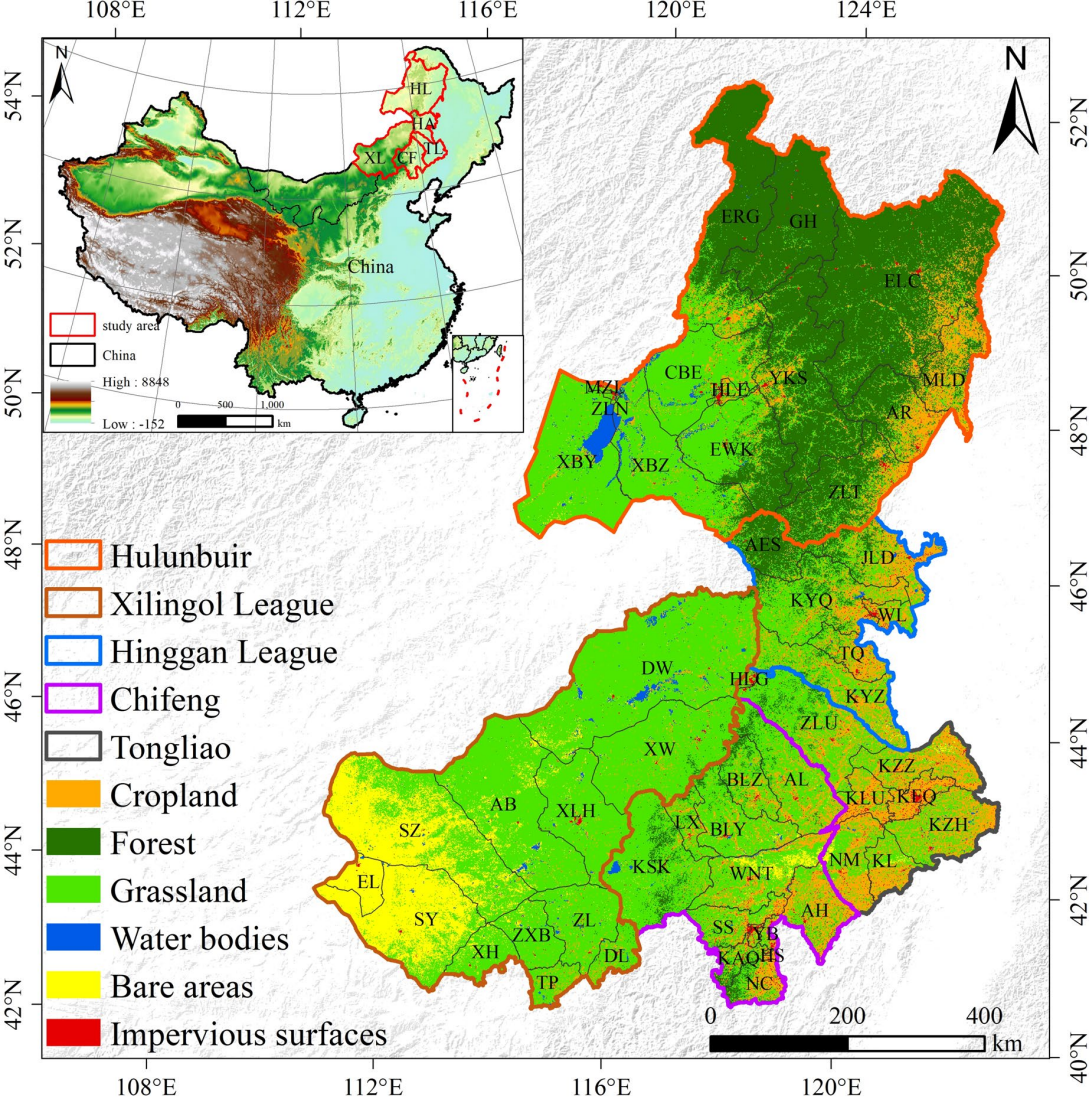
Land cover in EIM in 2020. NOTE: HL, Hulunbuir; HA, Hinggan League; TL, Tongliao; CF, Chifeng; XL, Xilingol League; AL, ArHorqin; KYZ, Horqin Right Wing Middle; TQ, Tuquan; KSK, Keshenketeng; KEQ, Horqin; YKS, Yakeshi; KYQ, Horqin Right Wing Front; YB, Yuanbaoshan; AB, Abaga Banner; KZH, Horqin Northeast; ERG, Ergun; NC, Ningcheng; AES, Aershan; SZ, Sonid Left Banner; HLG, Holingola; KL, Kulun; DL, Duolun County; ZLU, Jarud; HS, HongShan; LX, Linxi; XLH, Xilinhot; XBY, New Barag West; SY, Sonid Right Banner; XH, Xianghuang; CBE, Prairie Chenbarhu; XW, West Ujimqin; AR, Arun; KAQ, Harqin; BLZ, Balinzuo; ZL, Zhenglan; AH, Aohan; MZL, Manzhouli; SS, Songshan; KLU, Kailu; GH, Genhe; BLY, Balinyou; XBZ, New Barag East; EL, Erenhot; JLD, Jalaid; NM, Naiman; HLE, Hailar; EWK, Ewenki Autonomous; DW, East Ujimqin; MLD, Molidawadawoer; WL, UlanHot; ZLT, ZhaLanTun; ZXB, Zhengxiangbai; WNT, Wengniute; KZZ, Horqin East Middle; ELC, Oroqen Autonomous; ZLN, Jalainur; TP, Taipusi. Map created using ArcGIS 10.8 (Esri, https://www.esri.com).

The eastern region of Inner Mongolia (EIM) is located between 111°14′E–126°02′E and 42°12′N–53°18′N (Fig. 1). It comprises five administrative divisions: Hulunbuir, Tongliao, Chifeng, Hinggan League, and Xilingol League, covering about 6.5 × 10^5^ km^2^, which represents 56% of Inner Mongolia. The population was approximately 1.17 × 10⁷ in 2020. The terrain mainly consists of plateaus, plains, and basins, with elevations ranging from 300 to 2000 m. The region experiences a temperate continental monsoon climate, encompassing semi-humid, semi-arid, and arid regions, with mean annual precipitation ranging from 300 to 800 mm, which is mainly concentrated in summer and gradually decreases from east to west^25^. The administrative divisions of banners and counties form the third level of China’s administrative hierarchy, administered by prefecture-level divisions such as leagues or prefecture-level cities.

As a typical agro-pastoral ecotone, EIM has experienced increasing ecological pressure in recent decades. Population growth, rising food demand, climate change, and land use policy changes have accelerated LD^26^. In particular, the shift from traditional pastoralism to intensive farming has intensified both land reclamation and the abandonment of cultivated fields^27^. Therefore, identifying the dominant driving factors behind LD is critical for designing sustainable regional land use strategies.

## Materials and methods

### Data sources

#### Land cover data

In this study, we used the Global 30 m Land-Cover Dynamics Monitoring Product (GLC_FCS30D), developed by Liu et al. (available at 10.5281/zenodo.8239305) ^28^. This dataset was produced on Google Earth Engine platform, combining continuous change detection techniques with Landsat imagery. It has a spatial resolution of 30 m, includes 35 subclasses, and covers the period from 1985 to 2022. The dataset was updated every five years before 2000 and annually thereafter. The dataset has an overall classification accuracy of 80.88% for the basic land-cover system (10 classes) and has been used in various studies, including the assessment of forest loss, the characterisation of cropland spatiotemporal dynamics, and the analysis of construction land expansion.

In the present study, to be consistent with the acquisition of driver data, we used the land cover data from 1990, 2000, 2010 and 2020. Considering the land cover types in EIM, we reclassified the fine land-cover types into six categories: cropland, forest, grassland, water bodies, bare areas, and impervious surfaces. The classification results are presented in Table 1.

**Table 1:** Land cover classification and reclassification.

Major land-cover types	Fine land-cover types	Description	Merged Category
Cropland	Rainfed cropland	Land on which crops are grown	Cropland
	Herbaceous cover cropland		
	Irrigated cropland		
Forest	Closed evergreen broadleaved forest	Refers to the growth of trees, shrubs, bamboo, mangroves, and other forested areas	Forest
	Closed deciduous broadleaved forest		
	Open deciduous broadleaved forest		
	Closed evergreen needleleaved forest		
	Open evergreen needleleaved forest		
	Closed deciduous needleleaved forest		
	Open deciduous needleleaved forest		
	Open mixed-leaf forest		
Shrubland	Shrubland		
	Evergreen shrubland		
	Deciduous shrubland		
Grassland	Grassland	Describes grasslands dominated by herbaceous plants, covering more than 5%, including shrubland grassland mainly used for grazing and sparsely forested grassland with a canopy cover of less than 10%	Grassland
Bare areas	Sparse vegetation	Land not yet used, including difficult-to-use areas	
	Bare areas		Bare areas
	Unconsolidated bare areas		
Wetland	Swamp	Refers to natural land waters and water conservancy facilities	Water bodies
	Marsh		
	Flooded flat		
	Saline		
Water bodies	Water bodies		
Impervious surfaces	Impervious surfaces	Refers to urban and rural residential areas, as well as other industrial, mining, transportation and other land	Impervious surfaces

#### Land degradation drivers data

In the present study, we collected 12 indicators based on extensive literature^29,30^ and grouped them into four categories according to the characteristics of drivers (Table 2). The four groups are as follows: natural driver group, including annual average temperature and annual total precipitation; human activities driver group, including population density and sheep density; economic driver group, including the gross domestic product density of the primary industry, the gross domestic product density of the secondary industry, the gross domestic product density of the tertiary industry, and the total gross domestic product density; and urbanisation driver group, including distance to urban land, distance to rural settlements, distance to roads, and distance to other built-up land.

**Table 2:** Driving factors for LD.

Driver group	Data name	Unit	Year	Data sources	Orientation
Natural driver group	Annual average temperature	℃	1990, 2000, 2010, 2020	Climate Data Store (https://cds.climate.copernicus.eu/)	Higher values indicate a stronger influence on LD
	Annual total precipitation	mm	1990, 2000, 2010, 2020		Lower values indicate greater influence on LD. Direction adjusted using (1—normalised value)
Urbanisation driver group	Distance to roads	km	1990, 2000, 2010, 2020	Harvard Dataverse (https://dataverse.harvard.edu/), and Geofabrik Download Server (http://download.geofabrik.de/)	
	Distance to rural settlements	km	1990, 2000, 2010, 2020	Processed from the Chinese land–use/land-cover datasets produced by the Chinese Academy of Sciences (http://www.resdc.cn/)	
	Distance to urban land	km	1990, 2000, 2010, 2020		
	Distance to other built-up land	km	1990, 2000, 2010, 2020		
Human activities driver group	Population density	persons km⁻^2^	1990, 2000, 2010, 2020	Resource and Environment Science and Data Centre of China (http://www.resdc.cn/)	Higher values indicate a stronger influence on LD
	Sheep density	heads km⁻^2^	1990, 2000, 2010, 2017	Statistical Yearbook of Inner Mongolia, 1990–2020 (http://tj.nmg.gov.cn/)	
Economic driver group	Gross domestic product density of the primary industry (primary GDP density)	10^4^ CNY km⁻^2^	1992, 2000, 2010, 2020		
	Gross domestic product density of the secondary industry (secondary GDP density)	10^4^ CNY km⁻^2^	1992, 2000, 2010, 2020		
	Gross domestic product density of the tertiary industry (tertiary GDP density)	10^4^ CNY km⁻^2^	1992, 2000, 2010, 2020		
	Gross domestic product (GDP) density	10^4^ CNY km⁻^2^	1990, 2000, 2010, 2020		

Most driver data were available from 1990 to 2020, with the following exceptions: the gross domestic product densities of the primary, secondary, and tertiary industries were available from 1992, while sheep density data were available up to 2017. Data for natural drivers, urbanisation drivers, and population density were available up to 2020 (Fig. S1). For missing years, the most recent available data were used as proxies. Specifically, primary, secondary, and tertiary industry densities for 1990 were replaced with 1992 data, and sheep density for 2020 was represented by 2017 data. The annual average temperature and annual total precipitation were processed using Python. Urbanisation-related distances were calculated using the Euclidean distance tool in ArcGIS. Sheep numbers and economic data were obtained from the Inner Mongolia Statistical Yearbook, interpolated using Python with inverse distance weighting, and converted into density values. All driving factors were projected to WGS 1984 Albers and resampled to 1 × 1 km^2^ grids. The detailed sources and processing steps are presented in Table 2.

## Methods

### Evaluation of land cover change

The land cover transition matrix quantifies the transitions of area between different land cover types, thereby revealing the dynamics of land cover change. Specifically, it allows the quantification of transitions between two years, extracting the area of each type of land cover change, which helps to understand the spatial and temporal dynamics of land cover in EIM. Detailed descriptions of this method can be found in previous studies^31^. The calculation is as follows:

1 $${\mathrm{S}}_{{{\mathrm{ij}}}} { = }\left[ {\begin{array}{*{20}c} {\begin{array}{*{20}c} {{\mathrm{S}}_{{{11}}} } & {{\mathrm{S}}_{{{12}}} } \\ {{\mathrm{S}}_{{{21}}} } & {{\mathrm{S}}_{{{22}}} } \\ \end{array} } & {\begin{array}{*{20}c} \cdots & {{\mathrm{S}}_{{{\mathrm{1n}}}} } \\ \cdots & {{\mathrm{S}}_{{{\mathrm{2n}}}} } \\ \end{array} } \\ {\begin{array}{*{20}c} \cdots & \cdots \\ {{\mathrm{S}}_{{{\mathrm{n1}}}} } & {{\mathrm{S}}_{{{\mathrm{n2}}}} } \\ \end{array} } & {\begin{array}{*{20}c} \cdots & \cdots \\ \cdots & {{\mathrm{S}}_{{{\mathrm{nn}}}} } \\ \end{array} } \\ \end{array} } \right]$$

Here, S represents the area of each land cover class, n denotes the number of land cover types, and S_ij_ represents the area transitioning from land cover class i to class j.

### Analysis of land degradation and land restoration

#### Partial order theory

Partial Order Theory (POT) is a mathematical approach used to describe partial order relations among elements in a set. Unlike total order, partial order does not require all elements to be comparable, making it suitable for ranking objects characterised by multiple indicators, while retaining the information of all compared elements^32,33^.

A partial order relation must satisfy three fundamental properties^34^:

(1) Reflexivity: a ≤ a, for all a ∈ X;

(2) Transitivity: If a ≤ b and b ≤ c, then a ≤ c, for all a, b, c ∈ X;

(3) Antisymmetry: If a ≤ b and b ≤ a, then a = b, for all a, b ∈ X.

In this study, we applied POT to rank the drivers of LD in the EIM region during three periods: 1990–2000, 2000–2010, and 2010–2020. Here, the set X = {a, b, c, …} represents the banners/counties, and each element (e.g. a, b, c) is described by a group of LD drivers, such as population density and sheep density.

### Hasse Diagram Technique

The Hasse Diagram Technique (HDT) is a graphical method used to visualise partial order relations among objects^33^. Let X denote the finite set of objects, and F = {fi | i = 1, 2, …, |F|} denote the set of LD driver factors. Together, the objects and factors form a partially ordered set. In this study, X represents the 52 banners/counties in the EIM region. The study period is divided into three intervals: 1990–2000 (P1), 2000–2010 (P2), and 2010–2020(P3). The corresponding object sets are denoted as banners/counties_P1, banners/counties_P2, and banners/counties_P3. The factor set consists of 12 LD drivers, which are categorised into four groups: F_natural, F_human, F_economic, and F_urbanisation, forming a total of 12 partially ordered sets used for POT ranking and Hasse diagram visualisation.Comparisons between objects follow the rules specified below:

2 $$\left\{ {\begin{array}{*{20}c} {\begin{array}{*{20}c} {{\text{x }} \ge {\text{ y}}} & { < = > } & {f_{i} \left( {\mathrm{x}} \right){ } \ge { }f_{i} \left( {\mathrm{y}} \right){ }\begin{array}{*{20}c} \forall & {f_{i} { } \in {\text{ F}}} \\ \end{array} } \\ \end{array} } \\ {\begin{array}{*{20}c} {{\text{x }} \le {\text{ y}}} & { < = > } & {f_{i} \left( {\mathrm{x}} \right){ } \le f_{i} \left( {\mathrm{y}} \right)} \\ \end{array} { }\begin{array}{*{20}c} \forall & {f_{i} { } \in {\text{ F}}} \\ \end{array} } \\ {\begin{array}{*{20}c} {\text{x | y}} & {\text{ else}} \\ \end{array} } \\ \end{array} } \right.$$

When x ≥ y or x ≤ y, two objects are comparable. When x | y, the two objects are not comparable.

To ensure comparability, all input data were normalised to the interval [0, 1], using the following formula^35^:

3 $${\mathrm{fn}}_{{\mathrm{i}}} \left( {\mathrm{x}} \right){ = }\frac{{\left( {{\mathrm{f}}_{{\mathrm{i}}} \left( {\mathrm{x}} \right){\text{ - f}}_{{\mathrm{i}}} {\mathrm{min}}} \right)}}{{\left( {{\mathrm{f}}_{{\mathrm{i}}} {\text{max - f}}_{{\mathrm{i}}} {\mathrm{min}}} \right)}}$$

where fni is the value of the factor, and fimax and fimin are the maximum and minimum values, respectively.

The Hasse diagram is a directed graph^36^ that requires consistent orientation of all factors. In this study, orientation refers to the effect direction of each LD driver. For example, in the natural driver group, higher temperatures intensify LD, while increased precipitation mitigates it; precipitation values were adjusted using 1—fni(x) to ensure that larger values consistently indicate stronger impacts. In human activities, economic, and urbanisation groups, factors were oriented so that larger values correspond to stronger LD influence, maintaining consistency across all drivers.

County-level units were used as the basic administrative scale, and no thresholds or regression models were applied. It is important to note that this orientation step does not assume a linear relationship between drivers and LD. Rather, it is a directional standardization that allows different drivers to be compared in the partial-order ranking. Therefore, the 1—fni(x) adjustment does not alter the non-linear or threshold characteristics of the drivers within the POT-HDT framework.

### Application of the Hasse diagram technique

In this study, both the POT and HDT were applied to identify and analyse the dominant drivers of LD in EIM. The processing workflow and the application of HDT are described below.

**Fig. 2:**
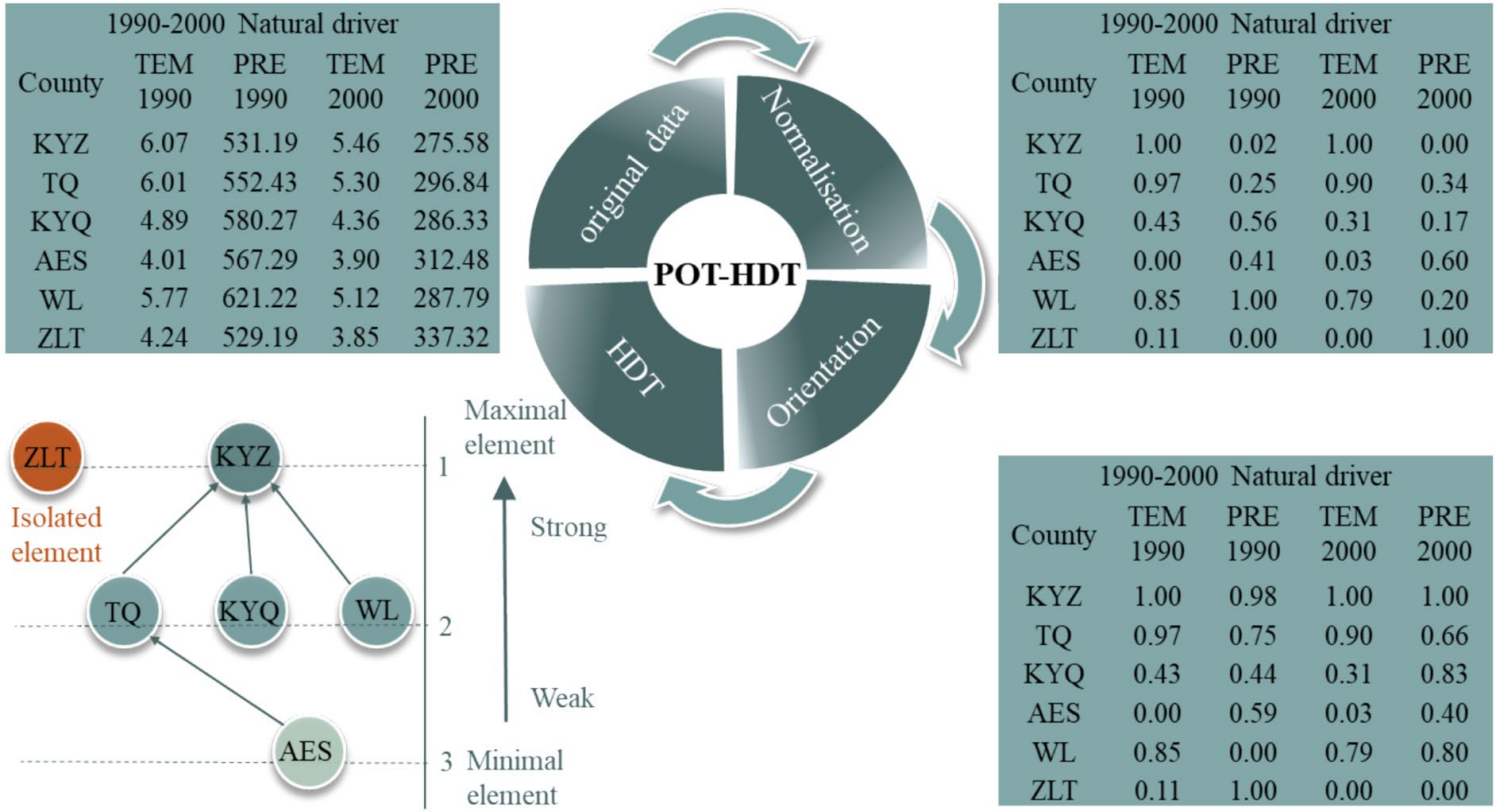
Application of POT and HDT for identifying the dominant LD drivers during 1990–2000.

(1) Data processing. The driving factors of LD in the EIM were extracted for three distinct time periods. Zonal statistics were used to calculate the average values of each factor at the county level. These average values were input into the POT to identify the dominant drivers of LD for each period (Fig. S2). Counties were classified based on these values to determine the dominant driver, which ensures reproducibility and provides a more precise scientific basis for policy-making by capturing local differences in driver influence.

(2) Normalisation and orientation. The data for each group of driving factors were normalised using Python. Both precipitation and urbanisation data were oriented to ensure consistency in the direction of all factors.

(3) Definition of Hasse diagram levels. In the Hasse diagram, the levels represent the relative positions of regions based on the influence of a given driver. Regions at higher levels are not dominated by others, indicating they are more strongly influenced. Regions at lower levels are dominated by more regions, signifying a weaker influence. The POT analysis and Hasse diagram construction were implemented in Python, and were used for ranking the drivers and visualizing the partial-order relationships.

(4) Application and case analysis. Figure 2 presents the dominant driving factors of LD in six counties of EIM during 1990–2000, based on the POT and HDT analyses. The diagram consists of three levels, ordered as follows: AES < {TQ, KYQ, WL} < KYZ. Three chains were identified: KYQ → KYZ, AES → TQ → KYZ, and WL → LYZ. Along these chains, the influence of natural drivers on LD increases progressively. KYZ is located at the highest level and is not dominated by any other county, indicating the strongest influence of natural factors. AES occupies the lowest level, dominated by the largest number of counties, indicating the weakest influence of natural factors. ZLT is an isolated element in the Hasse diagram because it exhibits extreme values in temperature and precipitation, making it incomparable with the other counties in the partial-order framework. In terms of regional land management, strategies should be tailored to the specific conditions of ZLT. In this structure, TQ and KYZ form a cover relation, meaning TQ is directly less than KYZ in the partial order, with no other county in between. Consequently, the influence of the driver on KYZ is directly greater than on TQ.

## Results

### Change in land degradation and its drivers

#### Analysis of land cover change from 1990 to 2020

**Fig. 3:**
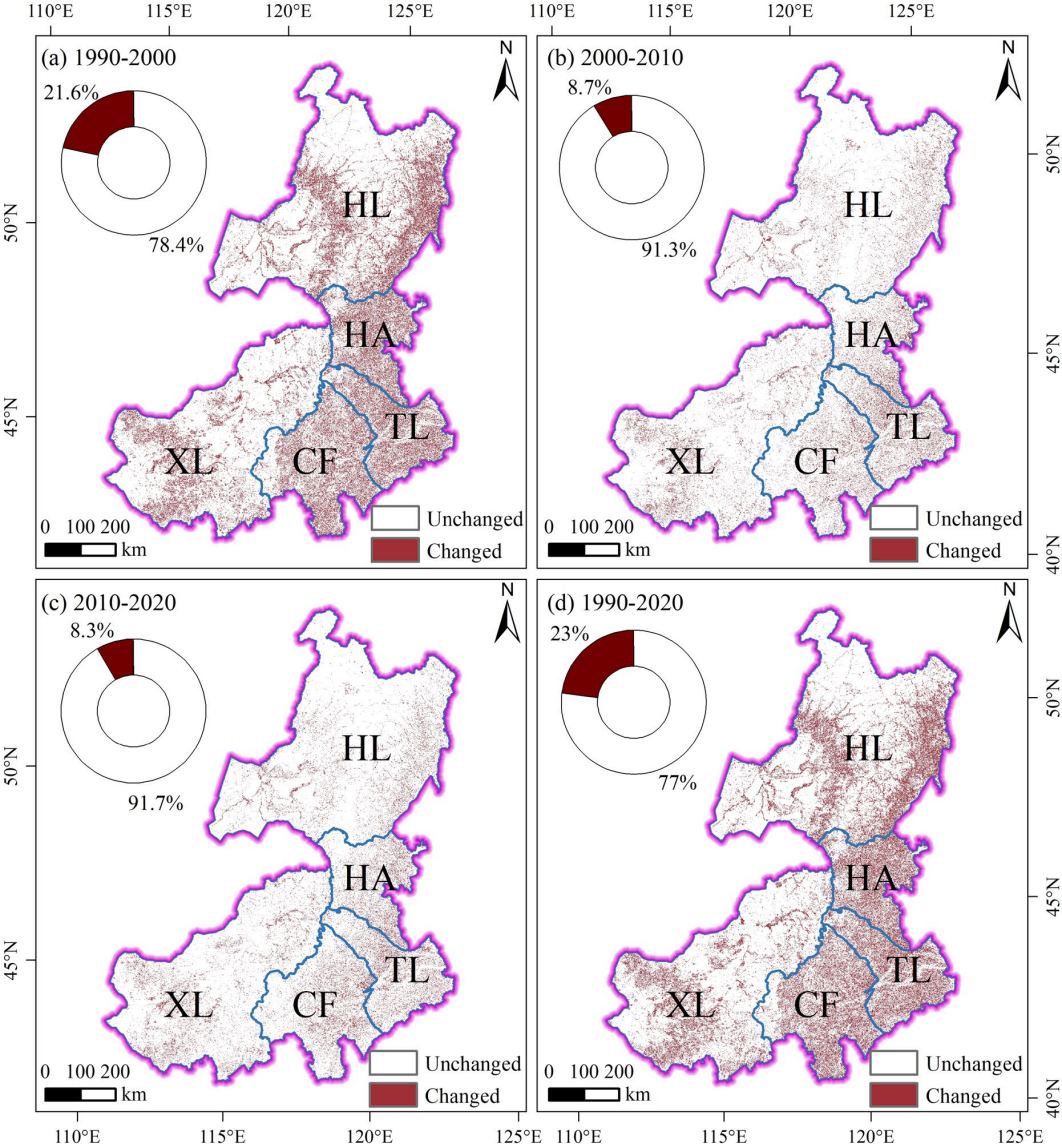
Spatiotemporal patterns of land cover changes in EIM (1990–2020): (**a**) changes during 1990–2000; (**b**) changes during 2000–2010; (**c**) changes during 2010–2020; (**d**) changes during 1990–2020. Map created using ArcGIS 10.8 (Esri, https://www.esri.com).

During 1990–2000, approximately 21.6% of the land in EIM underwent land cover change (Fig. 3a). Forest experienced the largest decrease (− 0.57%), primarily converted to cropland (5.58 × 10^3^ km^2^) and grassland (1.91 × 10^4^ km^2^). Impervious surfaces showed the largest increase (0.35%), mainly derived from cropland (1.36 × 10^3^ km^2^) and grassland (7.22 × 10^2^ km^2^) (Table 3; Table S1 and S2; Fig. S3). During 2000–2010, approximately 8.7% of the land in EIM underwent land cover change (Fig. 3b). Cropland experienced the largest decline (–0.27%), predominantly converted to grassland (1.74 × 10^4^ km^2^). Impervious surfaces increased by 0.21%, mainly sourced from cropland (7.03 × 10^2^ km^2^) and grassland (6.18 × 10^2^ km^2^) (Table 3; Table S1 and S3; Fig. S3). During 2010–2020, approximately 8.3% of the land in EIM underwent land cover change (Fig. 3c). Grassland experienced the largest decrease (–1.59%), primarily converted to cropland (1.94 × 10^4^ km^2^). Cropland exhibited the greatest expansion (0.89%), mainly derived from grassland (Table 3; Table S1, S4; Fig. S3). Over the entire period 1990–2020, nearly 1.49 × 10^5^ km^2^ of land in EIM underwent land cover change, accounting for about 23% of the study area (Fig. 3d).

**Table 3:** Major land cover changes in EIM across three time periods (1990–2000, 2000–2010, and 2010–2020).

Period	Most decreased type	Loss (area, %)	Main transitions (area, km^2^)	Most increased type	Gain (area / %)	Main sources (area, km^2^)
1990–2000	Forest	− 0.57	Cropland (5.58 × 10^3^), Grassland (1.91 × 10^4^)	Impervious surfaces	0.35	Cropland (1.36 × 10^3^), Grassland (7.22 × 10^2^)
2000–2010	Cropland	− 0.27	Grassland (1.74 × 10^4^)	Impervious surfaces	0.21	Cropland (7.03 × 10^2^), Grassland (6.18 × 10^2^)
2010–2020	Grassland	− 1.59	Cropland (1.94 × 10^4^)	Cropland	0.89	Grassland (1.94 × 10^4^)

### Spatiotemporal dynamics of land degradation and land restoration

**Fig. 4:**
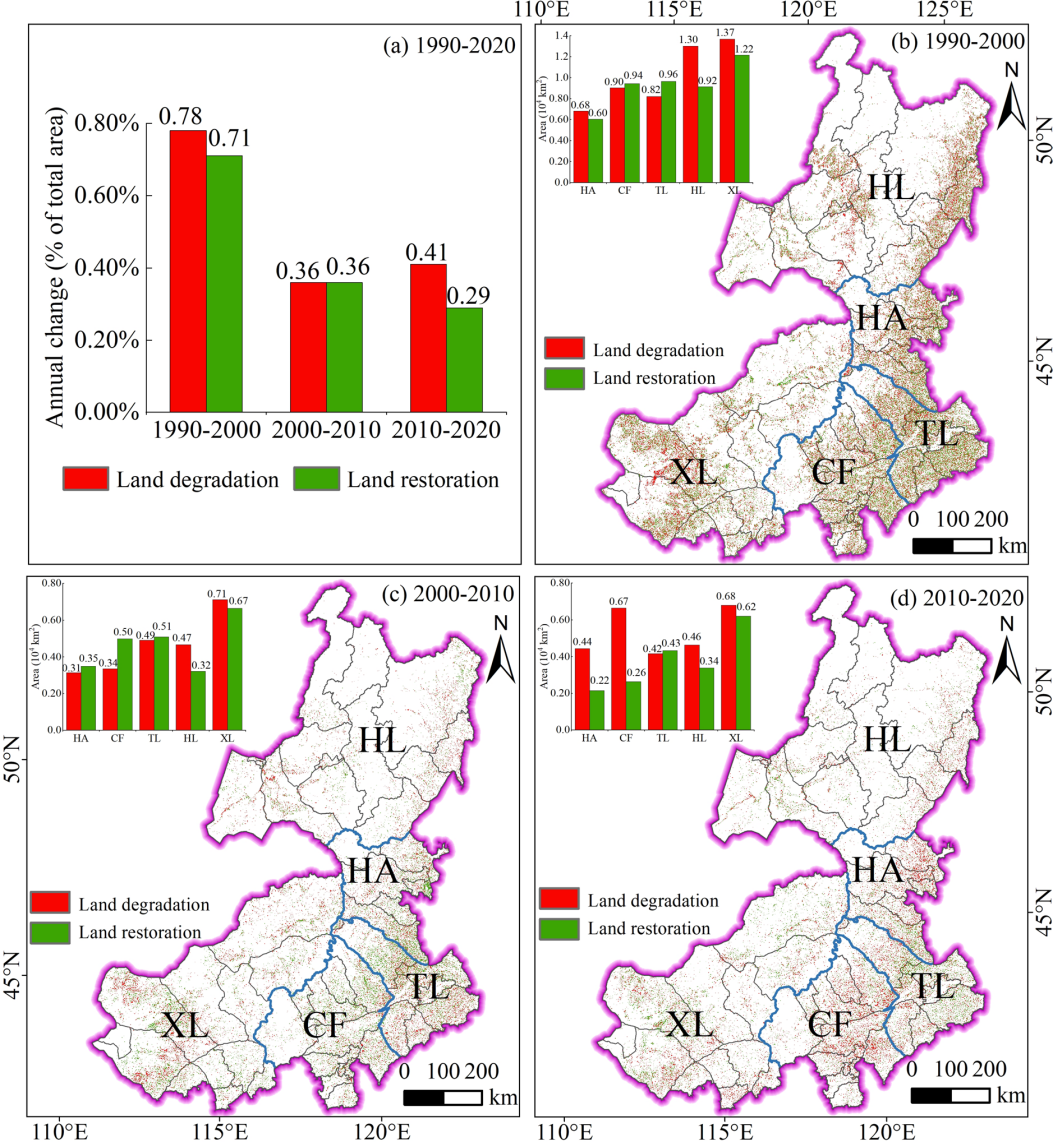
Spatiotemporal dynamics of land degradation and land restoration in EIM from 1990 to 2020: (**a**) annual change rate of LD and LR across the three periods; (**b**) LD and LR during 1990–2000; (**c**) LD and LR during 2000–2010; (**d**) LD and LR during 2010–2020. Map created using ArcGIS 10.8 (Esri, https://www.esri.com).

During 1990–2000, the annual change rate of LD was 0.78% (Fig. 4a), with LD primarily occurring in Hulunbuir and Xilingol (Fig. 4b). The annual restoration rate was 0.71%, with restoration mainly occurring in Xilingol. During 2000–2010, the annual change rate of LD decreased to 0.36% (Fig. 4a), and the degradation area decreased across all leagues (Fig. 4c). The annual restoration rate also dropped to 0.36%, with a decrease in restoration area across all regions. During 2010–2020, the annual change rate of LD increased to 0.41% (Fig. 4a). LD areas increased in Hinggan and Chifeng (Fig. 4d). The annual restoration rate dropped to 0.29%, with the restoration area decreasing in Hinggan, Chifeng, and Tongliao, while the restoration area increased in Hulunbuir. Over the 1990–2020 period, the net land degradation area was 5.83 × 10^4^ km^2^, while the net land restoration area was 4.62 × 10^4^ km^2^.

### Dominant drivers in each league/city during the period of 1990–2000

**Fig. 5:**
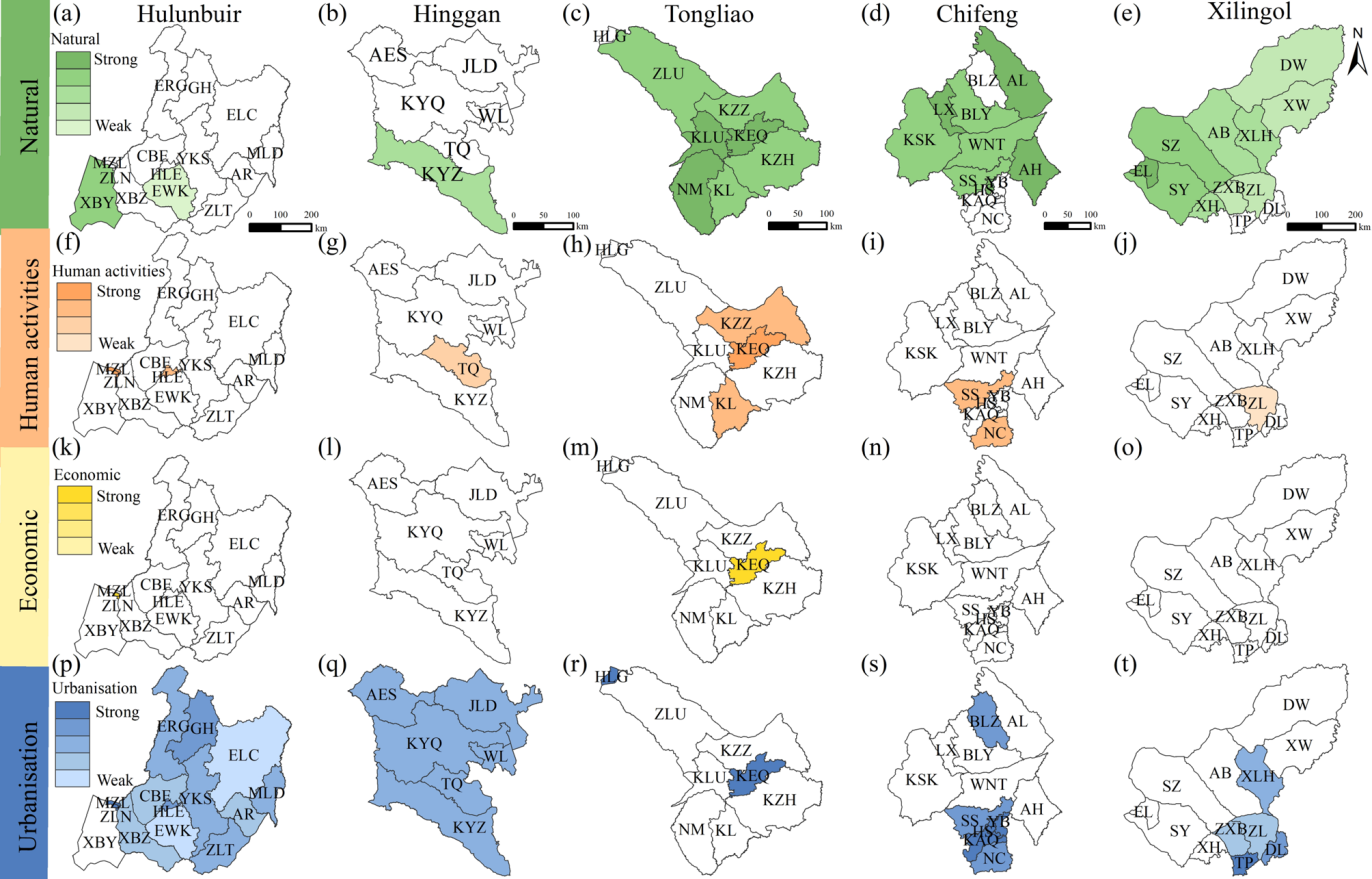
Dominant drivers of LD in EIM during 1990–2000. (**a**–**e**) Natural: Hulunbuir, Hinggan, Tongliao, Chifeng, and Xilingol; (**f**–**j**) Human activities; (**k**–**o**) Economic; (**p**–**t**) Urbanisation. Map created using ArcGIS 10.8 (Esri, https://www.esri.com).

Figure 5 presents the dominant drivers of LD in the EIM during 1990–2000, based on the partial order ranking of the drivers (Table S5 and Fig. S4). Figure 5a indicate that Hulunbuir was mainly affected by urbanisation (Table S6). Economic drivers had the least impact. Except for XBY, all counties and banners were influenced by urbanisation. XBY in the west and EWK in the centre were mainly influenced by natural drivers. In Hinggan, all counties and banners were influenced by urbanisation. The southern part of KYZ was also influenced by natural drivers (Fig. 5b and Table S6). Tongliao was mainly influenced by natural drivers. Except for HLG, all counties and banners were affected by natural factors (Fig. 5c and Table S6). Human activities were the second driver. They were found in the central and southern parts, including KZZ, KEQ, and KL. KEQ was influenced by natural, human activities, economic, and urbanisation drivers together. In Chifeng, natural drivers had the strongest impact. Urbanisation was the second, while economic drivers were not dominant (Fig. 5d and Table S6). In Xilingol, natural drivers had the largest impact. Urbanisation was the second. Human activities had smaller impacts, and they were mainly found in the southern part of ZL (Fig. 5e and Table S6).

### Dominant drivers in each league/city during the period of 2000–2010

**Fig. 6:**
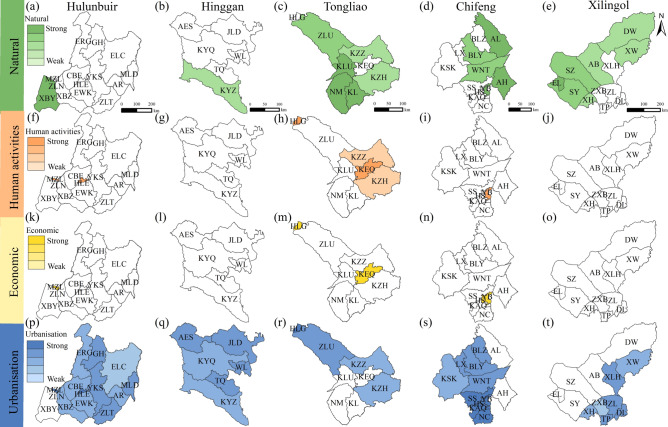
Dominant drivers of LD in EIM during 2000–2010. (**a**–**e**) Natural: Hulunbuir, Hinggan, Tongliao, Chifeng, and Xilingol; (**f**–**j**) Human activities; (**k**–**o**) Economic; (**p**–**t**) Urbanisation. Map created using ArcGIS 10.8 (Esri, https://www.esri.com).

Figure 6 presents the dominant drivers of LD in the EIM during 2000–2010, based on the partial order ranking of the drivers (Table S7 and Fig. S5). Hulunbuir was mainly affected by urbanisation (Fig. 6a and Table S8). Compared with the previous stage, the influence of natural drivers became weaker, as seen in the south of EWK. The influence of economic drivers became stronger in MZL. In Hinggan, urbanisation was still the main driver, the same as in the previous stage. The impact of human activities became weaker (Fig. 6b and Table S8). Tongliao was mainly affected by natural drivers. Compared with the previous stage, the influence of natural drivers became weaker in KEQ. The impact of urbanisation became stronger in the east and north. Human activities and economic drivers both became stronger (Fig. 6c and Table S8). In Chifeng, urbanisation was the main driver. Compared with the previous stage, its impact became much stronger, especially in the central and eastern parts. The impact of natural drivers became weaker in the central and western parts. The impact of human activities became weaker, while the influence of economic drivers became stronger (Fig. 6d and Table S8). In Xilingol, natural and urbanisation drivers were the main factors. The influence of natural drivers became weaker in the south (ZXB and ZL) and in the east (XLH). The impact of urbanisation became stronger (Fig. 6e and Table S8).

### Dominant drivers in each league/city during the period of 2010–2020

**Fig. 7:**
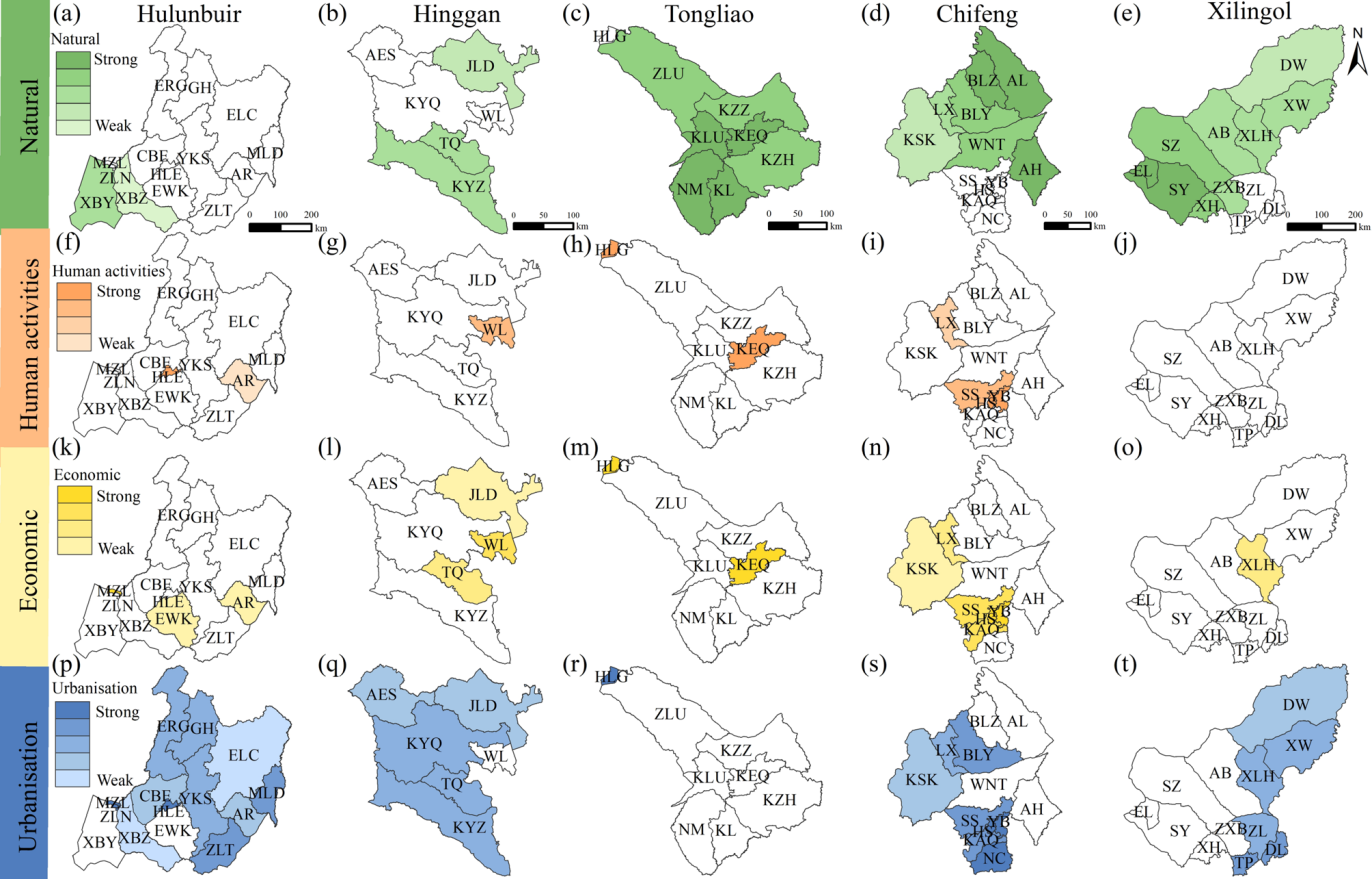
Dominant drivers of LD in EIM during 2010–2020. (**a**–**e**) Natural: Hulunbuir, Hinggan, Tongliao, Chifeng, and Xilingol; (**f**–**j**) Human activities; (**k**–**o**) Economic; (**p**–**t**) Urbanisation. Map created using ArcGIS 10.8 (Esri, https://www.esri.com).

Figure 7 presents the dominant drivers of LD in the EIM during 2010–2020, based on the partial order ranking of the drivers (Table S9 and Fig. S6). Hulunbuir was mainly affected by urbanisation (Fig. 7a and Table S10). Compared with the previous stage, the impact of urbanisation became weaker in the south (EWK). The influence of natural drivers became stronger in the west (XBZ). In Hinggan, the influence of urbanisation became weaker. Natural drivers became stronger in TQ and JLD (Fig. 7b and Table S10). Tongliao was more affected by natural drivers. The influence of human activities became weaker in KZH and KZZ. The impact of urbanisation also became weaker in the north and east (ZLU, KZZ and KZH) (Fig. 7c and Table S10). In Chifeng, the influence of natural drivers became stronger in the north. The impact of human activities became stronger in the central and northern areas, including HS, LX and SS. The effect of urbanisation became weaker in the central part (Fig. 7d and Table S10). In Xilingol, natural drivers were the main factor, followed by urbanisation (Fig. 7e and Table S10).

### Dominant drivers in EIM during the period of 1990–2020

**Fig. 8:**
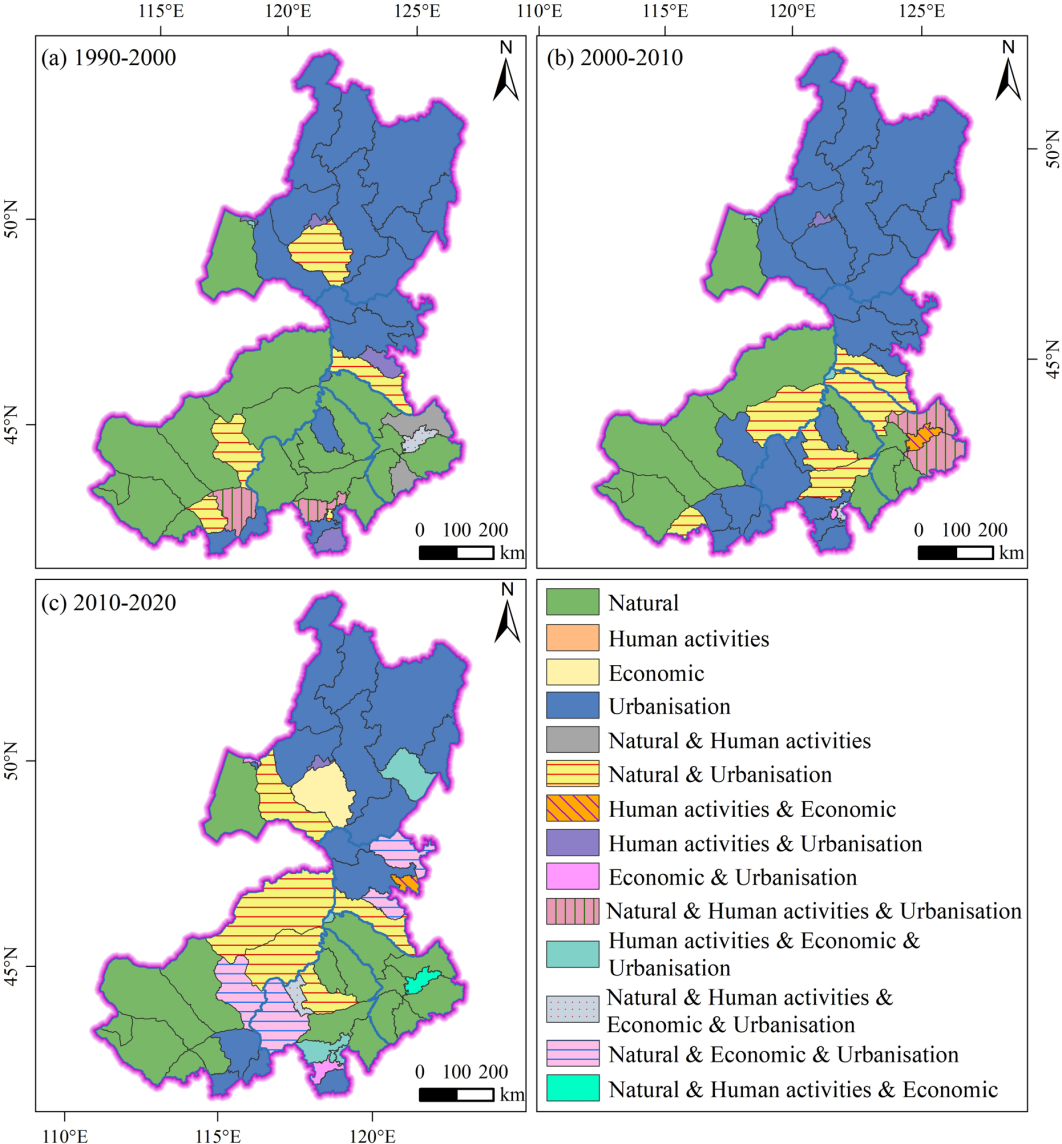
Dominant drivers of LD in EIM during1990–2020. (**a**) dominant drivers of LD during 1990–2000; (**b**) dominant drivers of LD during 2000–2010; (**c**) dominant drivers of LD during 2010–2020. Map created using ArcGIS 10.8 (Esri, https://www.esri.com).

Table 4 presents the counts of dominant driver groups for each league. For each league, the value indicates the number of counties dominated by a specific driver group during the respective period. During 1990–2000, the dominant driver in the north of the EIM was urbanisation, while natural drivers were dominant in the central and western parts (Fig. 8a). Among all counties and banners, 32 were mainly affected by urbanisation, 28 by natural drivers, 10 by human activities, and 2 by economic drivers (Table 4). The overall order of dominant drivers in this stage was: urbanisation > natural > human activities > economic. During 2000–2010, the influence of urbanisation expanded further. Urbanisation was the dominant driver in the north and central parts, while natural drivers dominated in the west and southeast (Fig. 8b). In total, 40 counties and banners were dominated by urbanisation, 20 by natural drivers, 8 by human activities, and 6 by economic drivers (Table 4). Compared with the previous stage, the impacts of urbanisation, natural, and economic drivers all became stronger. The dominant drivers followed the same order as in 1990–2000. During 2010–2020, urbanisation was the dominant driver in the north and central regions, while natural drivers dominated in the west and east (Fig. 8c). In this period, 32 counties and banners were mainly affected by urbanisation, 28 by natural drivers, 9 by human activities, and 15 by economic drivers (Table 4). Compared with the previous stage, the effects of urbanisation drivers became weaker, whereas the effects of natural, economic, and human activity drivers became stronger. The overall order of dominant drivers in this stage was: urbanisation > natural > economic > human activities. In the three periods of 1990–2000 (P1), 2000–2010 (P2), and 2010–2020 (P3), the dominant driver in Hulunbuir and Hinggan was urbanisation, although its influence gradually weakened (Table 4). The dominant driver in Tongliao and Xilingol was generally natural factors. Notably, the influence of urbanisation in Xilingol increased during P2. In Chifeng, natural factors were the dominant drivers in P1, whereas urbanisation became the dominant driver in P2 and P3.

**Table 4:** Ranking of driver groups by period (P1: 1990–2000, P2: 2000–2010, P3: 2010–2020).

League	Period	Natural	Human activities	Economic	Urbanisation
Hulunbuir	P1	2	3	1	13
	P2	1	3	2	13
	P3	2	2	3	12
Hinggan	P1	1	1	0	6
	P2	1	0	0	6
	P3	3	1	3	5
Tongliao	P1	7	3	1	2
	P2	6	4	2	4
	P3	7	2	2	1
Chifeng	P1	8	2	0	6
	P2	5	1	2	10
	P3	7	4	6	8
Xilingol	P1	10	1	0	5
	P2	7	0	0	7
	P3	9	0	1	6

## Discussion

From 1990 to 2020, LD and LR in EIM exhibited marked spatiotemporal variability. Overall, LD rates initially declined and then increased, whereas LR rates showed a continuous decrease. The sustained decline in LR can be attributed to limitations in ecological restoration policies. Although initiatives such as the Grain for Green program and grazing bans were implemented, their effectiveness was constrained by incomplete enforcement, insufficient resettlement of ecological migrants, and ongoing pressures from urbanisation and agricultural expansion. In addition, natural factors such as prolonged droughts and reduced precipitation limited vegetation recovery, particularly in areas with fragile soils and low water retention capacity. LD in EIM was influenced not only by climate change but also by urbanisation, agricultural expansion, and other human activities. Significant differences in land cover changes were observed among counties: in Hinggan League, LD was primarily driven by urbanisation; in Chifeng, it was influenced by both natural and urbanisation; in Tongliao, natural factors, particularly climate conditions affecting vegetation growth, were dominant; in Hulunbuir, urbanisation played a key role, mainly through cropland and built-up land expansion; and in Xilingol League, both natural factors and urbanisation were important drivers. Overall, urbanisation, population growth, cropland–grassland conversion, and climate change remain the main drivers of LD across the entire EIM region.

Previous studies provide useful context for our findings. Zhao et al.^37^ used remote sensing indices and the SDG15.3 framework to assess LD in Inner Mongolia. They identified cropland expansion as a major driver of LD in EIM, which is consistent with our results. Yuan et al.^38^ evaluated land degradation neutrality (LDN) using land use/cover, land productivity, and carbon stock indicators. Their results showed a decrease in restored land and an increase in degraded land. Although their study covered a wider area, the overall trends are in line with our observations in EIM. Lyu et al.^39^ integrated field measurements with vegetation and soil characteristics to establish a standard for regional monitoring of grassland degradation. They found that increased precipitation promoted the recovery of degraded grasslands, while high grazing intensity could worsen degradation, highlighting the important roles of both natural factors and human activities as drivers. By integrating subregional analyses with an overview of the entire study area, this study provides a comprehensive scientific basis for regional land management and policy planning.

### Urbanisation as the dominant driver of land degradation in EIM

Between 1990 and 2020, urbanisation was the primary driver of LD in EIM; however, its impact varied significantly across regions and stages. This study found that urbanisation exerted the most substantial influence on Hulunbuir and Hinggan throughout all periods. In contrast, its dominant role in Chifeng was most pronounced during stages P2 and P3. For Xilingol, stage P2 was characterized by the combined effects of natural factors and urbanisation (Table 4).

In Hulunbuir and Hinggan, where urbanisation was the dominant factor, economic growth and urban expansion gradually increased population density in cities and counties. This heightened demand for housing, subsequently promoting commercial land development and the expansion of built-up areas^40^. First, the urbanisation rate increased significantly, reaching 75.76% and 55.82% in 2020, respectively. Continuous government investment in infrastructure—such as transportation, education, and healthcare—improved urban living conditions, attracting a large number of rural residents to cities. Second, resource exploitation was a key driving force. The implementation of the Western Development Strategy accelerated the industrialization of Hulunbuir and Hinggan by facilitating the development of abundant coal and wind energy resources, which significantly sped up urbanisation. By comparison, urbanisation’s leading role in Chifeng was mainly observed in stages P2 and P3, with its urbanisation rate growing from 26.4% in 2000 to 51.2% in 2020. For Xilingol, stage P2 was influenced by both natural and urbanisation. This study further reveals that the impact of urbanisation there was concentrated in the agro-pastoral ecotones of the southern and eastern parts, areas which are more sensitive to anthropogenic disturbance.

The rapid urbanisation process has directly and indirectly led to LD. Directly, large-scale urban expansion has not only encroached upon grassland and cropland but has also led to regional landscape fragmentation^29^. This fragmentation trend has weakened the integrity and stability of grassland ecosystems. Urban expansion has reduced the connectivity of grassland spatial patterns, limiting the recovery of natural vegetation and accelerating LD by disrupting ecological processes. Dependent on industrial development, energy production and mineral resource extraction (particularly coal mining to meet urban demands) have resulted in vegetation destruction and waste accumulation. Indirectly, Zhou et al.^41^ found that urban expansion in the Hulunbuir grasslands accelerated grassland reclamation and increased population size. This in turn exacerbated water resource consumption and leading to irreversible LD. Coal mining in particular has not only directly destroyed large areas of grassland but has also led to the depletion and contamination of surface and groundwater in surrounding areas^42^. Therefore, urbanisation has exacerbated LD in multiple ways.

Furthermore, the rapid urbanisation process has to some extent weakened the effectiveness of national ecological protection policies aimed at restoring grassland ecosystems^43^. Zhu et al.^44^ found that while policies such as the Grain for Green program and grazing bans have alleviated pressure on grasslands, they have failed to effectively address the economic issues of these regions. Overgrazing has not been fundamentally curbed, and the resettlement of ecological migrants remains inadequate, limiting the development of livestock farming.

It is noteworthy that EIM contains several nationally protected forest and wetland reserves. These areas protected by strict laws and regulations, can potentially mitigate the LD directly caused by urban expansion. The study by Łągiewska et al.^45^ effectively demonstrates how to systematically integrate legal protection and spatial planning constraints into the decision-making process. They incorporated indicators such as forest coverage, the proportion of water bodies and wetlands, and impervious surface area into a multi-criteria decision analysis to quantify the spatial distribution of ecologically sensitive areas and guide management strategies. Drawing on this approach, future research in EIM could adopt a similar regional sensitivity algorithm. Such an algorithm could incorporate protected area boundaries as critical constraints into the formulation of urban and land management strategies. For instance, around the Hulun Lake National Nature Reserve, urban expansion and mining activities should be subject to stricter limitations.

### Natural factors as the dominant driver of land degradation in EIM

This study indicates that LD in Tongliao was primarily driven by natural factors across all three phases. In Xilingol, LD was mainly influenced by natural factors during P1 and P3. For Chifeng, natural factors were the dominant cause of LD only in P1. From 1990 to 2020, a long-term warming and drying trend was observed, characterized by a gradual increase in mean temperature and an overall decrease in annual precipitation. Against this background, short-term climate anomalies, such as consecutive dry years or periods of insufficient rainfall, likely exacerbated soil aridity. This in turn negatively affected agricultural production and grassland growth, potentially intensifying local LD^46^.

Chifeng, Tongliao, and Xilingol are situated in the agro-pastoral ecotone, a critical transitional zone from agricultural areas to grassland steppes in northern China. In this region, cropland and grassland overlap spatially. The complex land-use patterns, combined with climate variability and a fragile environment, make this area highly susceptible to climate change^47^. The soil texture in this ecotone is typically loose, resulting in a vulnerable land surface structure. This is characterized by low vegetation cover and poor soil water retention capacity. Consequently, even minor disturbances can easily damage the ecosystem. Research by Wang et al. [61] suggests that intensive cultivation and overgrazing in this area can amplify the effects of climatic stressors such as drought, high evaporation, low precipitation, and strong winds, thereby contributing to LD.

Between 1990 and 2020, grassland area in Chifeng, Tongliao, and Xilingol decreased by 3357 km^2^, a change closely associated with climate change. Chen et al.^48^ found that both precipitation and temperature are key factors affecting vegetation growth. Firstly, under reduced precipitation, grassland plants often limit stomatal opening to reduce water loss, which restricts photosynthesis and inhibits growth. Secondly, rising temperatures may promote photosynthesis but can also increase evapotranspiration and reduce water availability, potentially increasing drought risk under certain conditions. However, Bartold et al.^49^ found that the actual effect of drought on vegetation in Poland grasslands strongly depends on topography. Grasslands in river valleys or near urban areas can maintain stable growth for up to two weeks under high temperatures by adapting to reduced water availability. This indicates that their impact on LD is highly heterogeneous at local scales and does not necessarily cause large-scale degradation. This suggests that alongside climatic stress human activities are equally important. Studies have shown that mowing intensity can lead to species-poor grasslands and changes vegetation productivity, and that the complex grassland pattern is strongly influenced by historical and current land use practice^50^. Liu et al.^51^ noted that the grassland carrying capacity in the northern part of the agro-pastoral zone is low, primarily due to reduced precipitation and higher temperatures, which increase surface evapotranspiration, decrease soil moisture, and further hinder vegetation growth, leading to grassland degradation. Dong et al.^52^ pointed out that in arid regions with significant precipitation fluctuations, herders often struggle to adjust livestock numbers in time to match grassland carrying capacity, which further intensifies grassland degradation.

### Recommendations for future sustainable development in EIM cities

The dominant drivers of LD vary significantly among the EIM cities, and therefore future strategies should be tailored accordingly. In Hulunbuir, urbanisation is the dominant driver. Policies should focus on precise industrial and urban area zoning, the designation of restricted development zones in ecologically sensitive areas, and the promotion of clean-energy agriculture combined with multi-functional integration of agriculture, forestry, and pasture, which balances production with ecological restoration to reduce further degradation. In Hinggan, urbanisation is the dominant driver. Measures should include strict zoning in urban expansion areas, the establishment of green belts and ecological buffer zones around cities to limit grassland conversion, low-intensity sustainable agriculture, high-frequency remote sensing monitoring to ensure policy enforcement, and the continuation of programs returning farmland to forest and grassland to improve ecological quality^53^. In Tongliao, natural factors are the dominant driver of LD. Priority should be given to ecological restoration, including afforestation, grassland reseeding, soil water retention techniques, and rotational grazing to reduce overgrazing pressure. In Chifeng, LD is driven by both natural factors and urbanisation. Policies should combine ecological restoration with control of urban expansion, including afforestation and grassland restoration in affected areas, the implementation of a drought early warning system based on the vegetation stress index, and strict control over the conversion of cropland and ecological land to construction land. In Xilingol, LD is influenced by both natural and urbanisation. Afforestation and grassland restoration should be carried out in the western and northern regions affected by climate, sustainable pastoral management should be promoted in the southern agro-pastoral areas, and strict water resource protection measures should be implemented in arid and semi-arid regions. Integrating the dominant drivers identified by the POT-HDT model with zoning controls and targeted measures can improve regional development management^54^ (Table 5).

**Table 5:** POT-HDT identified dominant drivers and targeted policy measures in EIM cities.

League	Dominant driver	Main policy measures
Hulunbuir	Urbanisation	1. Industrial and urban area zoning and restricted development zones 2. Clean-energy agriculture and multi-functional integration of agriculture, forestry, and pasture
Hinggan	Urbanisation	1. Strict zoning in urban expansion areas 2. Establish green belts and ecological buffers around cities 3. Low-intensity sustainable agriculture with high-frequency remote sensing monitoring 4. Continue returning farmland to forest and grassland
Tongliao	Natural factors	1. Afforestation and grassland reseeding 2. Soil water retention techniques 3. Rotational grazing to reduce overgrazing
Chifeng	Natural factors and urbanisation	1. Afforestation and grassland restoration in affected areas 2. Drought early warning system 3. Strict control of conversion of farmland and ecological land to construction land
Xilingol	Natural factors and urbanisation	1. Afforestation and grassland restoration in western and northern regions 2. Sustainable pastoral management in southern agro-pastoral areas 3. Strict water resource protection in arid and semi-arid regions

### Advantages and limitations of POT

Understanding the drivers of LD is crucial for comprehending the evolution of land systems, curbing the degradation process, and formulating effective governance and restoration strategies^55^. In this study, we applied the POT and HDT to analyse the dominant drivers of LD at the county level in EIM. The use of POT and HDT not only improved the accuracy of the analysis but also provided a scientific basis for formulating more effective land management strategies. Unlike regression, machine learning, or Geographical Detector methods, which have limitations such as linear assumptions, high data requirements, or inability to handle multi-factor interactions, POT-HDT can simultaneously account for nonlinear, hierarchical, and interdependent relationships among drivers, consider their dynamic characteristics, and reveal dominance relationships using only a small amount of data. This makes POT-HDT particularly suitable for identifying key LD drivers at administrative scales and for supporting sustainable land management policies. Compared to our previous study in the Hohhot–Baotou–Ordos region^56^, which focused on grassland conversion out (GCO) in a highly urbanised and economically developed area, the present study examines LD across multiple land cover types, including grassland, forest, and water bodies, in EIM. EIM is an ecologically fragile and resource-rich region. The methods (POT-HDT) and the indicators are the same as in the previous study. However, this study is different in several key ways. First, it covers a longer period from 1990 to 2020. Second, before 2010, human activities had a greater effect on LD than economic factors. These differences show the specific mechanisms of LD in this region. They also provide a scientific basis for sustainable land management in EIM. EIM is a key area for cross-border ecological and economic interactions between China, Mongolia, and Russia.

However, several limitations should be acknowledged. The POT-HDT framework has inherent methodological constraints: rankings are sensitive to input data and indicator selection, particularly in the presence of extreme values or strongly nonlinear effects; applying POT-HDT to other regions or datasets may produce different results, reflecting limitations in model transferability; and the framework does not inherently provide statistical significance testing. We did not perform formal sensitivity analyses in this study, so the robustness of the rankings under alternative data treatments or normalization methods has not been directly tested. Future studies could address this by testing alternative normalization methods or excluding incomplete variables, which would help further assess the reliability of the results.

Data-related limitations also exist. The aggregation of pixel-level LD data to the county scale may mask local hotspots or introduce statistical artifacts due to the modifiable areal unit problem (MAUP). Future analyses at finer grid scales (1 km × 1 km) could help validate our findings and capture fine-scale heterogeneity more precisely. Reclassifying 35 land-cover subclasses into six broad categories may obscure some ecological nuances, such as differences between irrigated and rainfed croplands. The GLC_FCS30D dataset, with its high spatial and temporal resolution, is suitable for large-scale monitoring of land cover dynamics and has been widely validated in northern China. By comparing multiple land cover products, we further confirmed that the GLC_FCS30D data for EIM are reliable. However, it should be noted that the current definition of LD in this study is based solely on land-cover transitions. This approach enables the analysis of broad spatial and temporal patterns of LD across the study area, but it may overlook degradation within a single land-cover type, such as declining grassland productivity, shrub encroachment, or thinning in forests, which do not result in a change of land-cover class. Consequently, LD rates may be underestimated, and this proxy does not provide direct validation of soil or vegetation productivity.

Some limitations are related to data access. While most driver datasets span 1990–2020, some exceptions exist, such as sheep density (2017) and industry density (1992). Proxy data were used for missing years to maintain temporal continuity, which may introduce some uncertainty, particularly regarding livestock patterns and climate anomalies in the final years. The study period ends in 2020 primarily due to the availability of consistent long-term driver datasets. Policies and environmental changes in EIM after 2020 may influence the relative importance of drivers. Therefore, the results of this study may not fully reflect recent dynamics. Topographic variables, including DEM, slope, and aspect, are important factors in LD studies, but they were not included in the current analysis due to limited availability of consistent data for all study years. However, these variables are relatively stable over time. Urbanisation was represented using distance-based metrics to urban land, rural settlements, roads, and other built-up areas, providing a practical proxy for urbanisation intensity. Nevertheless, the choice of indicators may still affect the relative ranking of drivers, and distance-based metrics cannot fully capture the complexity of urbanisation, such as variations in land-use intensity and infrastructure development. Consequently, the influence of urbanisation on LD may be partially underestimated or overestimated. Across the three study periods (P1, P2, and P3), the Pearson correlation coefficients between the economic and urbanisation indicators were all below 0.7. According to Dormann et al.^57^, this threshold indicates the point at which collinearity begins to severely distort model estimation. Therefore, no severe collinearity exists between the two groups, and the ranking of driver groups in the Hasse diagram is unlikely to be affected. These two sets of indicators capture different aspects of human activities, reducing potential multicollinearity and ensuring that the Hasse diagram levels reflect distinct dimensions of human influence on LD.

Recent advances in artificial intelligence (AI) have demonstrated strong potential for environmental modelling in data-scarce regions. For instance, hybrid AI frameworks combining Long Short-Term Memory (LSTM), eXtreme Gradient Boosting (XGBoost), and clustering techniques have been successfully applied for interpretable water quality prediction and spatial pattern analysis in the Niger Delta^58^. Machine learning approaches integrating Support Vector Machine (SVM), Random Forest, and LSTM, coupled with explainable AI tools, have enabled accurate prediction and driver identification for shoreline change and erosion^59^. In addition, morphometric analysis combined with machine learning has enhanced flood management and catchment prioritisation in regions with sparse environmental data^60^. These studies highlight that AI-based modelling in data-scarce contexts can provide interpretable, data-driven insights and may improve the reliability of LD research in future studies.

Future research will address these limitations through multiple pathways: adopting finer-grained land cover classifications to better capture ecological distinctions; conducting analyses at finer grid scales (1 km × 1 km) to validate findings and capture fine-scale heterogeneity; incorporating the missing years’ data to improve temporal completeness; integrating continuous vegetation indices (normalized difference vegetation index, net primary productivity) to capture intra-class productivity declines and provide a more comprehensive assessment of LD; incorporating DEM and derived topographic variables to complement the current analysis; adding additional urbanisation indicators (built-up area, nighttime light data) to more accurately characterise urbanisation pressures; and comparing POT-HDT results with alternative methods (including machine learning approaches) to further evaluate the robustness and reliability of driver rankings.

## Conclusions

This study developed a framework that combines LD analysis with POT and the HDT. It was used to identify and rank the drivers of LD in EIM from 1990 to 2020. The results show that approximately 23% of the land experienced cover change this time. Both the rates of LD and LR have been decreasing over time. From 1990 to 2000, the main land changes were a reduction in forest and an increase in impervious surfaces. Between 2000 and 2010, the predominant changes were a decrease in cropland and an increase in impervious surfaces. From 2010 to 2020, the main changes were a reduction in grassland and an increase in cropland. Twelve drivers were grouped into four categories: natural, human activity, economic, and urbanisation. In the first two periods, the order of influence was: urbanisation > natural > human activities > economic. In the third period, the order changed to: urbanisation > natural > economic > human activities. Over time, the influence of human activities gradually weakened, whereas the role of economic drivers became stronger. The findings of this study provide a foundation for future research on land use and land management in EIM. By identifying the dominant drivers and their temporal shifts, this work can guide targeted monitoring, policy design, and restoration planning, and inform efforts to achieve LDN and sustainable land use goals at regional scales.

The application of POT and HDT complements traditional statistical and machine learning approaches by providing better interpretability, transparency, and visualisation in analysing multi–factor interactions. These methods provide a robust framework for analysing the priority and spatial heterogeneity of LD drivers, supporting evidence–based land management in arid and semi–arid regions. Future research could extend this framework to multi–scenario simulations, spatial planning, and restoration effectiveness assessments, contributing to LDN and the achievement of the SDG.

## Supplementary Information

Supplementary Information.

## Data Availability

The author confirms that all data generated or analysed during this study are included in this published article.
