# Supplementary material for: Ranking land degradation drivers in eastern Inner Mongolia using partial order theory and Hasse diagram analysis
Source: Sci Rep. 2026 Apr 10;16:16762. doi: 10.1038/s41598-026-47280-5 (PMC13222888; doi:10.1038/s41598-026-47280-5)
Supplement: Supplementary file 1 — Supplementary Information. [file 41598_2026_47280_MOESM1_ESM.docx]

**Ranking Land Degradation Drivers in eastern Inner Mongolia Using Partial Order Theory and Hasse Diagram Analysis**

**Kaixin Liu^1^, Batunacun^1, 2, *^, Yong Mei^1^, Yu Feng^1^, Ruifang Guo^1^, Chang An^1^, Yaxin Wang^3, 4^, Bin Sun^3^**

^1^ College of Geographical Science, Inner Mongolia Normal University, Hohhot 010028, China

^2^ State Key Laboratory of resources Environmental, Institute of Geographic Sciences and Natural Resources Research, Chinese Academy of Sciences, Beijing 100101, China

^3^ Institute of Forest Resource Information Techniques, Chinese Academy of Forestry, Beijing 100091, China

^4^ Key Laboratory of Forestry Remote Sensing and Information System, NFGA, Beijing 100091, China

^*^E-mail addresses: 20210041@imnu.edu.cn

**Table S**

| Land cover type | 1990-2000 | 2000-2010 | 2010-2020 | 1990-2020 |
| --- | --- | --- | --- | --- |
| Cropland | 0.07% | -0.27% | 0.89% | 0.70% |
| Forest | -0.57% | -0.01% | 0.32% | -0.26% |
| Grassland | -0.23% | 0.09% | -1.59% | -1.74% |
| Water body | 0.13% | -0.04% | 0.05% | 0.14% |
| Bare areas | 0.25% | 0.02% | 0.15% | 0.42% |
| Impervious surface | 0.35% | 0.21% | 0.19% | 0.74% |

**Table S1.** Net chang of land cover change from 1990 to 2020.

|  |  | 2000 | | | | | | |
| --- | --- | --- | --- | --- | --- | --- | --- | --- |
|  |  | Cropland | Forest | Grassland | Water body | Bare areas | Impervious surface | losses |
| 1990 | Cropland | 49926 | 3643 | 32352 | 1160 | 1036 | 1360 | 39551 |
|  | Forest | 5576 | 136497 | 19107 | 198 | 4 | 77 | 24962 |
|  | Grassland | 33130 | 17400 | 281292 | 1837 | 10385 | 722 | 63474 |
|  | Water body | 736 | 203 | 1622 | 4602 | 100 | 22 | 2683 |
|  | Bare areas | 592 | 7 | 8899 | 363 | 36038 | 50 | 9911 |
|  | Impervious surface | 0 | 0 | 0 | 0 | 0 | 1589 | 0 |
|  | gains | 40034 | 21253 | 61980 | 3558 | 11525 | 2231 | 140581 |

**Table S2****.** Land cover transition matrix in eastern Inner Mongolia from 1990 to 2000.

|  |  | 2010 | | | | | | |
| --- | --- | --- | --- | --- | --- | --- | --- | --- |
|  |  | Cropland | Forest | Grassland | Water body | Bare areas | Impervious surface | losses |
| 2000 | Cropland | 70147 | 888 | 17447 | 434 | 341 | 703 | 19813 |
|  | Forest | 1066 | 152960 | 3631 | 67 | 19 | 7 | 4790 |
|  | Grassland | 16186 | 3763 | 317631 | 548 | 4526 | 618 | 25641 |
|  | Water body | 591 | 86 | 588 | 6701 | 179 | 15 | 1459 |
|  | Bare areas | 213 | 0 | 4539 | 125 | 42649 | 37 | 4914 |
|  | Impervious surface | 0 | 0 | 0 | 0 | 0 | 3820 | 0 |
|  | gains | 18056 | 4737 | 26205 | 1174 | 5065 | 1380 | 56617 |

**Table S3.** Land cover transition matrix in eastern Inner Mongolia from 2000 to 2010.

|  |  | 2020 | | | | | | |
| --- | --- | --- | --- | --- | --- | --- | --- | --- |
|  |  | Cropland | Forest | Grassland | Water body | Bare areas | Impervious surface | losses |
| 2010 | Cropland | 73010 | 1057 | 12959 | 374 | 269 | 534 | 15193 |
|  | Forest | 1089 | 154244 | 2269 | 74 | 6 | 15 | 3453 |
|  | Grassland | 19392 | 4366 | 313738 | 638 | 5077 | 625 | 30098 |
|  | Water body | 344 | 70 | 381 | 6925 | 138 | 17 | 950 |
|  | Bare areas | 175 | 12 | 4119 | 200 | 43169 | 39 | 4545 |
|  | Impervious surface |  |  |  |  |  | 5200 | 0 |
|  | gains | 21000 | 5505 | 19728 | 1286 | 5490 | 1230 | 54239 |

**Table S4.** Land cover transition matrix in eastern Inner Mongolia from 2010 to 2020.

| County | TM1990 | PRE_1990 | TM_2000 | PRE_2000 | PN_1990 | Sh_1990 | PN_2000 | Sh_2000 | Dy_1992 | De_1992 | Ds_1992 | GDP_1990 | Dy_2000 | De_2000 | Ds_2000 | GDP_2000 | Cs_1990 | Nc_1990 | Dl_1990 | Kq_1990 | Cs_2000 | Nc_2000 | Dl_2000 | Kq_2000 |
| --- | --- | --- | --- | --- | --- | --- | --- | --- | --- | --- | --- | --- | --- | --- | --- | --- | --- | --- | --- | --- | --- | --- | --- | --- |
| AL | 0.83 | 0.53 | 0.71 | 0.66 | 0.08 | 0.73 | 0.06 | 0.40 | 0.06 | 0.00 | 0.00 | 0.02 | 0.04 | 0.00 | 0.00 | 0.01 | 0.44 | 0.93 | 0.59 | 0.47 | 0.44 | 0.94 | 0.58 | 0.47 |
| KYZ | 0.77 | 0.26 | 0.61 | 0.65 | 0.06 | 0.46 | 0.05 | 0.38 | 0.06 | 0.00 | 0.00 | 0.02 | 0.05 | 0.00 | 0.00 | 0.01 | 0.44 | 0.93 | 0.83 | 0.56 | 0.44 | 0.95 | 0.56 | 0.61 |
| TQ | 0.76 | 0.20 | 0.58 | 0.57 | 0.21 | 0.68 | 0.14 | 0.40 | 0.14 | 0.00 | 0.00 | 0.05 | 0.25 | 0.01 | 0.00 | 0.05 | 0.48 | 0.96 | 0.80 | 0.54 | 0.48 | 0.98 | 0.66 | 0.72 |
| KSK | 0.61 | 0.49 | 0.45 | 0.37 | 0.05 | 0.40 | 0.04 | 0.27 | 0.03 | 0.00 | 0.00 | 0.01 | 0.03 | 0.00 | 0.00 | 0.01 | 0.36 | 0.87 | 0.56 | 0.60 | 0.36 | 0.86 | 0.71 | 0.60 |
| KEQ | 0.93 | 0.42 | 0.80 | 0.56 | 0.54 | 1.00 | 0.44 | 1.00 | 0.61 | 0.02 | 0.01 | 0.36 | 1.00 | 0.10 | 0.06 | 0.42 | 0.66 | 0.97 | 0.92 | 0.04 | 0.65 | 1.00 | 0.90 | 0.07 |
| YKS | 0.24 | 0.28 | 0.30 | 0.41 | 0.09 | 0.02 | 0.06 | 0.01 | 0.02 | 0.00 | 0.00 | 0.03 | 0.03 | 0.00 | 0.00 | 0.02 | 0.70 | 0.57 | 0.79 | 0.69 | 0.70 | 0.69 | 0.81 | 0.68 |
| KYQ | 0.60 | 0.12 | 0.42 | 0.61 | 0.09 | 0.45 | 0.06 | 0.31 | 0.05 | 0.00 | 0.00 | 0.02 | 0.07 | 0.00 | 0.00 | 0.02 | 0.39 | 0.89 | 0.73 | 0.79 | 0.40 | 0.86 | 0.70 | 0.88 |
| YB | 0.97 | 0.28 | 0.90 | 0.49 | 0.56 | 0.56 | 0.45 | 0.35 | 0.33 | 0.03 | 0.01 | 0.14 | 0.45 | 0.27 | 0.11 | 0.77 | 0.68 | 0.99 | 0.88 | 0.99 | 0.68 | 1.00 | 0.96 | 0.99 |
| AB | 0.34 | 0.86 | 0.08 | 0.86 | 0.00 | 0.24 | 0.00 | 0.18 | 0.01 | 0.00 | 0.00 | 0.00 | 0.02 | 0.00 | 0.00 | 0.00 | 0.05 | 0.56 | 0.00 | 0.60 | 0.05 | 0.33 | 0.34 | 0.60 |
| KZH | 0.96 | 0.29 | 0.81 | 0.39 | 0.11 | 0.67 | 0.10 | 0.42 | 0.15 | 0.00 | 0.00 | 0.04 | 0.18 | 0.00 | 0.01 | 0.04 | 0.52 | 0.95 | 0.79 | 0.00 | 0.52 | 0.95 | 0.69 | 0.00 |
| ERG | 0.16 | 0.36 | 0.26 | 0.53 | 0.02 | 0.03 | 0.02 | 0.02 | 0.01 | 0.00 | 0.00 | 0.01 | 0.02 | 0.00 | 0.00 | 0.00 | 0.75 | 0.70 | 0.66 | 0.70 | 0.75 | 0.78 | 0.54 | 0.69 |
| NC | 1.00 | 0.14 | 1.00 | 0.50 | 0.47 | 0.56 | 0.35 | 0.55 | 0.25 | 0.01 | 0.00 | 0.10 | 0.35 | 0.02 | 0.02 | 0.11 | 0.71 | 0.97 | 0.79 | 0.91 | 0.71 | 0.99 | 0.91 | 0.91 |
| AES | 0.48 | 0.16 | 0.34 | 0.50 | 0.03 | 0.03 | 0.02 | 0.01 | 0.02 | 0.00 | 0.00 | 0.01 | 0.02 | 0.00 | 0.00 | 0.01 | 0.46 | 0.22 | 0.74 | 0.91 | 0.46 | 0.22 | 0.75 | 0.91 |
| SZ | 0.48 | 0.94 | 0.25 | 0.89 | 0.00 | 0.14 | 0.00 | 0.11 | 0.00 | 0.00 | 0.00 | 0.00 | 0.01 | 0.00 | 0.00 | 0.00 | 0.00 | 0.00 | 0.10 | 0.55 | 0.00 | 0.00 | 0.00 | 0.54 |
| HLG | 0.54 | 0.38 | 0.36 | 0.61 | 0.27 | 0.46 | 0.30 | 0.56 | 0.12 | 0.03 | 0.01 | 0.17 | 0.25 | 0.09 | 0.07 | 0.33 | 0.89 | 0.94 | 0.39 | 1.00 | 0.89 | 0.96 | 0.92 | 1.00 |
| KL | 1.00 | 0.28 | 0.91 | 0.41 | 0.12 | 0.63 | 0.09 | 0.46 | 0.09 | 0.00 | 0.00 | 0.03 | 0.11 | 0.00 | 0.00 | 0.03 | 0.61 | 0.97 | 0.66 | 0.34 | 0.61 | 0.98 | 0.64 | 0.33 |
| DL | 0.40 | 0.72 | 0.19 | 0.32 | 0.09 | 0.65 | 0.06 | 0.38 | 0.07 | 0.00 | 0.00 | 0.02 | 0.11 | 0.00 | 0.00 | 0.03 | 0.74 | 0.91 | 0.69 | 0.33 | 0.74 | 0.93 | 0.77 | 0.32 |
| ZLU | 0.82 | 0.34 | 0.68 | 0.69 | 0.06 | 0.56 | 0.05 | 0.33 | 0.06 | 0.00 | 0.00 | 0.02 | 0.10 | 0.00 | 0.00 | 0.02 | 0.52 | 0.93 | 0.64 | 0.53 | 0.52 | 0.94 | 0.66 | 0.52 |
| HS | 1.00 | 0.39 | 0.91 | 0.51 | 0.58 | 0.29 | 0.47 | 0.21 | 0.36 | 0.05 | 0.05 | 0.03 | 0.50 | 0.11 | 0.16 | 0.61 | 0.88 | 1.00 | 0.99 | 0.97 | 0.88 | 0.97 | 0.98 | 0.97 |
| LX | 0.67 | 0.55 | 0.53 | 0.39 | 0.22 | 0.58 | 0.16 | 0.40 | 0.15 | 0.00 | 0.00 | 0.04 | 0.19 | 0.01 | 0.01 | 0.06 | 0.65 | 0.98 | 0.83 | 0.67 | 0.65 | 0.97 | 0.90 | 0.67 |
| XLH | 0.42 | 0.78 | 0.19 | 0.78 | 0.03 | 0.31 | 0.03 | 0.18 | 0.03 | 0.01 | 0.00 | 0.01 | 0.03 | 0.02 | 0.00 | 0.04 | 0.27 | 0.65 | 0.56 | 0.81 | 0.28 | 0.77 | 0.80 | 0.81 |
| XBY | 0.18 | 0.52 | 0.00 | 0.99 | 0.00 | 0.18 | 0.00 | 0.17 | 0.01 | 0.00 | 0.00 | 0.00 | 0.02 | 0.00 | 0.00 | 0.00 | 0.20 | 0.23 | 0.52 | 0.59 | 0.20 | 0.29 | 0.29 | 0.59 |
| SY | 0.60 | 0.91 | 0.41 | 0.73 | 0.01 | 0.16 | 0.01 | 0.13 | 0.01 | 0.00 | 0.00 | 0.00 | 0.01 | 0.00 | 0.00 | 0.00 | 0.23 | 0.43 | 0.53 | 0.79 | 0.23 | 0.53 | 0.53 | 0.79 |
| XH | 0.49 | 0.75 | 0.29 | 0.62 | 0.02 | 0.46 | 0.01 | 0.36 | 0.02 | 0.00 | 0.00 | 0.01 | 0.03 | 0.00 | 0.00 | 0.00 | 0.57 | 0.53 | 0.60 | 0.62 | 0.57 | 0.69 | 0.53 | 0.61 |
| CBE | 0.10 | 0.31 | 0.24 | 0.44 | 0.01 | 0.16 | 0.01 | 0.14 | 0.02 | 0.00 | 0.00 | 0.01 | 0.02 | 0.00 | 0.00 | 0.01 | 0.48 | 0.69 | 0.55 | 0.76 | 0.48 | 0.73 | 0.66 | 0.75 |
| XW | 0.44 | 0.60 | 0.22 | 0.61 | 0.01 | 0.44 | 0.01 | 0.30 | 0.03 | 0.00 | 0.00 | 0.00 | 0.04 | 0.00 | 0.00 | 0.01 | 0.35 | 0.74 | 0.55 | 0.83 | 0.35 | 0.73 | 0.45 | 0.83 |
| AR | 0.43 | 0.36 | 0.21 | 0.16 | 0.12 | 0.20 | 0.08 | 0.08 | 0.11 | 0.00 | 0.00 | 0.03 | 0.16 | 0.00 | 0.00 | 0.02 | 0.46 | 0.82 | 0.61 | 0.85 | 0.46 | 0.86 | 0.63 | 0.85 |
| KAQ | 0.94 | 0.39 | 0.88 | 0.35 | 0.42 | 0.53 | 0.30 | 0.34 | 0.16 | 0.01 | 0.00 | 0.08 | 0.14 | 0.01 | 0.01 | 0.06 | 0.73 | 0.99 | 0.90 | 0.92 | 0.73 | 0.99 | 0.93 | 0.92 |
| BLZ | 0.73 | 0.42 | 0.58 | 0.50 | 0.20 | 0.62 | 0.14 | 0.39 | 0.12 | 0.00 | 0.00 | 0.03 | 0.12 | 0.01 | 0.01 | 0.03 | 0.61 | 0.98 | 0.64 | 0.53 | 0.61 | 1.00 | 0.66 | 0.53 |
| ZL | 0.41 | 0.70 | 0.18 | 0.45 | 0.02 | 0.68 | 0.01 | 0.30 | 0.05 | 0.00 | 0.00 | 0.01 | 0.04 | 0.00 | 0.00 | 0.01 | 0.28 | 0.56 | 0.72 | 0.48 | 0.28 | 0.56 | 0.78 | 0.48 |
| AH | 0.97 | 0.20 | 0.92 | 0.55 | 0.22 | 0.47 | 0.17 | 0.32 | 0.14 | 0.00 | 0.00 | 0.04 | 0.19 | 0.01 | 0.01 | 0.04 | 0.58 | 0.98 | 0.81 | 0.76 | 0.58 | 0.99 | 0.86 | 0.76 |
| MZL | 0.01 | 0.58 | 0.06 | 0.93 | 0.90 | 0.85 | 0.80 | 0.40 | 0.45 | 0.08 | 0.04 | 0.70 | 0.43 | 0.11 | 0.25 | 0.79 | 0.81 | 0.82 | 1.00 | 0.95 | 0.81 | 0.87 | 1.00 | 0.95 |
| SS | 0.89 | 0.44 | 0.79 | 0.49 | 0.46 | 0.50 | 0.37 | 0.37 | 0.24 | 0.00 | 0.00 | 0.08 | 0.21 | 0.01 | 0.01 | 0.06 | 0.42 | 0.97 | 0.89 | 0.87 | 0.43 | 0.98 | 0.84 | 0.87 |
| KLU | 0.92 | 0.50 | 0.80 | 0.70 | 0.26 | 0.79 | 0.20 | 0.42 | 0.33 | 0.00 | 0.00 | 0.09 | 0.55 | 0.01 | 0.01 | 0.11 | 0.57 | 0.94 | 0.58 | 0.24 | 0.57 | 0.94 | 0.74 | 0.23 |
| GH | 0.27 | 0.35 | 0.27 | 0.42 | 0.12 | 0.00 | 0.09 | 0.00 | 0.00 | 0.00 | 0.00 | 0.01 | 0.01 | 0.00 | 0.00 | 0.01 | 0.69 | 0.42 | 0.71 | 0.93 | 0.69 | 0.46 | 0.87 | 0.93 |
| BLY | 0.79 | 0.43 | 0.68 | 0.59 | 0.06 | 0.54 | 0.05 | 0.32 | 0.05 | 0.00 | 0.00 | 0.01 | 0.05 | 0.00 | 0.00 | 0.01 | 0.48 | 0.98 | 0.54 | 0.57 | 0.48 | 0.99 | 0.77 | 0.57 |
| XBZ | 0.21 | 0.37 | 0.04 | 0.62 | 0.00 | 0.19 | 0.00 | 0.17 | 0.01 | 0.00 | 0.00 | 0.00 | 0.02 | 0.00 | 0.00 | 0.00 | 0.47 | 0.36 | 0.71 | 0.71 | 0.47 | 0.48 | 0.69 | 0.71 |
| EL | 0.60 | 1.00 | 0.43 | 1.00 | 0.03 | 0.02 | 0.09 | 0.27 | 0.00 | 0.00 | 0.00 | 0.00 | 0.00 | 0.00 | 0.01 | 0.02 | 0.49 | 0.59 | 0.79 | 0.89 | 0.49 | 0.66 | 0.82 | 0.89 |
| JLD | 0.61 | 0.09 | 0.41 | 0.62 | 0.12 | 0.45 | 0.09 | 0.28 | 0.06 | 0.00 | 0.00 | 0.03 | 0.15 | 0.00 | 0.00 | 0.03 | 0.53 | 0.94 | 0.51 | 0.69 | 0.53 | 0.96 | 0.66 | 0.77 |
| NM | 0.97 | 0.29 | 0.89 | 0.60 | 0.16 | 0.62 | 0.12 | 0.34 | 0.14 | 0.00 | 0.00 | 0.05 | 0.18 | 0.01 | 0.01 | 0.05 | 0.66 | 0.97 | 0.80 | 0.48 | 0.66 | 0.98 | 0.78 | 0.47 |
| HLE | 0.00 | 0.30 | 0.26 | 0.30 | 0.59 | 0.48 | 0.57 | 0.31 | 0.19 | 0.03 | 0.04 | 0.35 | 0.30 | 0.07 | 0.11 | 0.41 | 0.88 | 0.89 | 0.97 | 0.92 | 0.88 | 0.88 | 0.92 | 0.92 |
| EWK | 0.16 | 0.29 | 0.27 | 0.41 | 0.03 | 0.15 | 0.03 | 0.11 | 0.01 | 0.00 | 0.00 | 0.02 | 0.03 | 0.00 | 0.00 | 0.01 | 0.20 | 0.38 | 0.52 | 0.76 | 0.20 | 0.36 | 0.55 | 0.76 |
| DW | 0.36 | 0.51 | 0.14 | 0.72 | 0.00 | 0.24 | 0.00 | 0.22 | 0.01 | 0.00 | 0.00 | 0.00 | 0.02 | 0.00 | 0.00 | 0.00 | 0.08 | 0.53 | 0.23 | 0.65 | 0.08 | 0.52 | 0.47 | 0.65 |
| MLD | 0.30 | 0.31 | 0.02 | 0.15 | 0.09 | 0.17 | 0.07 | 0.06 | 0.08 | 0.00 | 0.00 | 0.04 | 0.13 | 0.00 | 0.00 | 0.02 | 0.57 | 0.89 | 0.65 | 0.87 | 0.57 | 0.92 | 0.71 | 0.87 |
| WL | 0.73 | 0.00 | 0.55 | 0.60 | 0.26 | 0.31 | 0.20 | 0.16 | 0.07 | 0.02 | 0.01 | 0.13 | 0.17 | 0.04 | 0.04 | 0.19 | 0.61 | 0.89 | 0.94 | 0.57 | 0.61 | 0.85 | 0.85 | 0.88 |
| ZLT | 0.51 | 0.27 | 0.33 | 0.40 | 0.12 | 0.16 | 0.08 | 0.09 | 0.06 | 0.00 | 0.00 | 0.03 | 0.08 | 0.00 | 0.00 | 0.02 | 0.65 | 0.76 | 0.51 | 0.85 | 0.65 | 0.75 | 0.63 | 0.85 |
| ZXB | 0.41 | 0.70 | 0.17 | 0.51 | 0.03 | 0.63 | 0.02 | 0.35 | 0.03 | 0.00 | 0.00 | 0.01 | 0.03 | 0.00 | 0.00 | 0.01 | 0.37 | 0.67 | 0.71 | 0.44 | 0.37 | 0.63 | 0.43 | 0.43 |
| WNT | 0.88 | 0.41 | 0.79 | 0.61 | 0.13 | 0.46 | 0.09 | 0.27 | 0.10 | 0.00 | 0.00 | 0.03 | 0.13 | 0.00 | 0.00 | 0.03 | 0.49 | 0.94 | 0.51 | 0.71 | 0.49 | 0.95 | 0.77 | 0.71 |
| KZZ | 0.90 | 0.34 | 0.74 | 0.53 | 0.16 | 0.72 | 0.09 | 0.43 | 0.25 | 0.00 | 0.00 | 0.06 | 0.17 | 0.00 | 0.01 | 0.03 | 0.64 | 0.96 | 0.76 | 0.10 | 0.64 | 0.97 | 0.78 | 0.17 |
| ELC | 0.22 | 0.24 | 0.08 | 0.00 | 0.04 | 0.01 | 0.02 | 0.00 | 0.01 | 0.00 | 0.00 | 0.01 | 0.01 | 0.00 | 0.00 | 0.00 | 0.35 | 0.42 | 0.57 | 0.73 | 0.35 | 0.55 | 0.31 | 0.73 |
| ZLN | 0.05 | 0.54 | 0.06 | 0.88 | 1.00 | 0.27 | 1.00 | 0.18 | 1.00 | 1.00 | 1.00 | 1.00 | 0.80 | 1.00 | 1.00 | 1.00 | 1.00 | 0.57 | 0.89 | 1.00 | 1.00 | 0.65 | 0.97 | 1.00 |
| TP | 0.36 | 0.60 | 0.10 | 0.40 | 0.21 | 0.61 | 0.12 | 0.31 | 0.11 | 0.00 | 0.00 | 0.03 | 0.17 | 0.01 | 0.01 | 0.05 | 0.70 | 0.99 | 0.86 | 0.24 | 0.70 | 1.00 | 0.84 | 0.23 |

**Table S5.** Input data for the Hasse diagram (1990-2000).

| League | County | Natural | Human activities | Economic | Urbanisation | Dominant driver | League | County | Natural | Human activities | Economic | Urbanisation | Dominant driver |
| --- | --- | --- | --- | --- | --- | --- | --- | --- | --- | --- | --- | --- | --- |
| Hulunbuir | YKS | 4 | 9 | 10 | 3 | Urbanisation | Hinggan | KYZ | 3 | 5 | 11 | 3 | Natural & Urbanisation |
|  | ERG | 4 | 11 | 16 | 3 | Urbanisation |  | TQ | 4 | 3 | 4 | 3 | Human activities & Urbanisation |
|  | XBY | 2 | 12 | 17 | 6 | Natural |  | KYQ | 4 | 6 | 11 | 3 | Urbanisation |
|  | CBE | 5 | 10 | 15 | 4 | Urbanisation |  | AES | 5 | 11 | 15 | 3 | Urbanisation |
|  | AR | 5 | 7 | 7 | 4 | Urbanisation |  | JLD | 4 | 6 | 7 | 3 | Urbanisation |
|  | MZL | 2 | 1 | 2 | 1 | Human activities & Urbanisation |  | WL | 4 | 4 | 4 | 3 | Urbanisation |
|  | GH | 4 | 7 | 16 | 2 | Urbanisation | Tongliao | KEQ | 1 | 1 | 1 | 1 | Natural & Human activities & Economic & Urbanisation |
|  | XBZ | 5 | 11 | 17 | 4 | Urbanisation |  | HLG | 3 | 2 | 3 | 1 | Urbanisation |
|  | HLE | 5 | 2 | 3 | 2 | Human activities & Urbanisation |  | KL | 2 | 2 | 8 | 3 | Natural & Human activities |
|  | EWK | 5 | 10 | 13 | 5 | Natural & Urbanisation |  | KZH | 2 | 3 | 6 | 3 | Natural |
|  | MLD | 6 | 8 | 7 | 3 | Urbanisation |  | ZLU | 2 | 6 | 10 | 3 | Natural |
|  | ZLT | 4 | 7 | 9 | 3 | Urbanisation |  | NM | 1 | 5 | 5 | 2 | Natural |
|  | ELC | 6 | 10 | 17 | 5 | Urbanisation |  | KZZ | 2 | 2 | 4 | 3 | Natural & Human activities |
|  | ZLN | 3 | 1 | 1 | 1 | Human activities & Economic & Urbanisation |  | KLU | 1 | 2 | 2 | 3 | Natural |
| Chifeng | AL | 1 | 3 | 12 | 4 | Natural | Xilingol | AB | 3 | 10 | 17 | 6 | Natural |
|  | KSK | 2 | 8 | 14 | 4 | Natural |  | SZ | 2 | 13 | 19 | 7 | Natural |
|  | YB | 2 | 2 | 2 | 1 | Urbanisation |  | DL | 4 | 4 | 9 | 2 | Urbanisation |
|  | NC | ISO | 2 | 3 | 2 | Human activities & Urbanisation |  | XLH | 3 | 9 | 5 | 3 | Natural & Urbanisation |
|  | HS | 1 | 3 | 2 | 1 | Natural & Urbanisation |  | SY | 2 | 11 | 18 | 5 | Natural |
|  | LX | 1 | 3 | 5 | 2 | Natural |  | XH | 3 | 6 | 15 | 4 | Natural |
|  | KAQ | 2 | 3 | 4 | 1 | Urbanisation |  | XW | 4 | 8 | 14 | 5 | Natural |
|  | BLZ | 3 | 4 | 7 | 2 | Urbanisation |  | ZL | 4 | 4 | 13 | 4 | Natural & Human activities & Urbanisation |
|  | AH | 1 | 4 | 6 | 2 | Natural |  | EL | 1 | 7 | 8 | 3 | Natural |
|  | SS | 2 | 2 | 4 | 2 | Natural & Human activities & Urbanisation |  | DW | 4 | 9 | 17 | 5 | Natural |
|  | BLY | 2 | 7 | 12 | 3 | Natural |  | ZXB | 4 | 5 | 14 | 4 | Natural & Urbanisation |
|  | WNT | 2 | 6 | 7 | 3 | Natural |  | TP | 5 | 4 | 6 | 1 | Urbanisation |

**Table S6.** Rankings and dominant drivers of LD in EIM for the period 1990–2000.

| County | TM_2000 | PRE_2000 | TM_2010 | PRE_2010 | PN_2000 | Sh_2000 | PN_2010 | Sh_2010 | Dy_2000 | De_2000 | Ds_2000 | GDP_2000 | Dy_2010 | De_2010 | Ds_2010 | GDP_2010 | Cs_2000 | Nc_2000 | Dl_2000 | Kq_2000 | Cs_2010 | Nc_2010 | Dl_2010 | Kq_2010 |
| --- | --- | --- | --- | --- | --- | --- | --- | --- | --- | --- | --- | --- | --- | --- | --- | --- | --- | --- | --- | --- | --- | --- | --- | --- |
| AL | 0.73 | 0.67 | 0.91 | 0.68 | 0.06 | 0.40 | 0.05 | 0.12 | 0.04 | 0.00 | 0.00 | 0.01 | 0.07 | 0.01 | 0.01 | 0.01 | 0.47 | 0.94 | 0.67 | 0.46 | 0.47 | 0.93 | 0.71 | 0.78 |
| KYZ | 0.63 | 0.68 | 0.71 | 0.55 | 0.05 | 0.38 | 0.04 | 0.13 | 0.05 | 0.00 | 0.00 | 0.01 | 0.07 | 0.00 | 0.00 | 0.01 | 0.54 | 0.94 | 0.65 | 0.60 | 0.63 | 0.90 | 0.83 | 0.64 |
| TQ | 0.59 | 0.59 | 0.70 | 0.53 | 0.14 | 0.40 | 0.12 | 0.13 | 0.25 | 0.01 | 0.00 | 0.05 | 0.26 | 0.01 | 0.01 | 0.02 | 0.54 | 0.96 | 0.67 | 0.72 | 0.72 | 0.95 | 0.79 | 0.50 |
| KSK | 0.43 | 0.39 | 0.51 | 0.33 | 0.03 | 0.27 | 0.03 | 0.07 | 0.03 | 0.00 | 0.00 | 0.01 | 0.04 | 0.01 | 0.00 | 0.01 | 0.41 | 0.81 | 0.71 | 0.60 | 0.42 | 0.83 | 0.77 | 0.73 |
| KEQ | 0.81 | 0.59 | 0.97 | 0.47 | 0.44 | 1.00 | 0.42 | 0.44 | 1.00 | 0.10 | 0.06 | 0.42 | 0.94 | 0.36 | 0.24 | 0.41 | 0.72 | 1.00 | 0.91 | 0.09 | 0.72 | 0.96 | 0.92 | 0.88 |
| YKS | 0.31 | 0.43 | 0.17 | 0.32 | 0.06 | 0.01 | 0.05 | 0.01 | 0.03 | 0.00 | 0.00 | 0.02 | 0.07 | 0.01 | 0.01 | 0.01 | 0.77 | 0.62 | 0.85 | 0.67 | 0.77 | 0.71 | 0.80 | 0.54 |
| KYQ | 0.41 | 0.62 | 0.49 | 0.47 | 0.05 | 0.31 | 0.04 | 0.11 | 0.07 | 0.00 | 0.00 | 0.02 | 0.10 | 0.00 | 0.00 | 0.01 | 0.42 | 0.85 | 0.65 | 0.85 | 0.56 | 0.84 | 0.75 | 0.47 |
| YB | 0.91 | 0.51 | 0.99 | 0.38 | 0.46 | 0.35 | 0.55 | 0.39 | 0.45 | 0.27 | 0.11 | 0.77 | 1.00 | 0.38 | 0.29 | 0.45 | 0.72 | 1.00 | 0.97 | 0.98 | 0.84 | 0.99 | 0.94 | 0.98 |
| AB | 0.10 | 0.84 | 0.60 | 0.81 | 0.00 | 0.18 | 0.00 | 0.03 | 0.02 | 0.00 | 0.00 | 0.00 | 0.01 | 0.00 | 0.00 | 0.00 | 0.22 | 0.22 | 0.45 | 0.61 | 0.24 | 0.46 | 0.24 | 0.67 |
| KZH | 0.81 | 0.39 | 0.94 | 0.00 | 0.10 | 0.42 | 0.09 | 0.17 | 0.18 | 0.00 | 0.01 | 0.04 | 0.15 | 0.01 | 0.01 | 0.02 | 0.58 | 0.94 | 0.71 | 0.00 | 0.45 | 0.96 | 0.77 | 0.71 |
| ERG | 0.27 | 0.50 | 0.16 | 0.55 | 0.01 | 0.02 | 0.01 | 0.01 | 0.02 | 0.00 | 0.00 | 0.00 | 0.02 | 0.00 | 0.00 | 0.00 | 0.64 | 0.58 | 0.38 | 0.68 | 0.54 | 0.47 | 0.55 | 0.62 |
| NC | 1.00 | 0.47 | 0.93 | 0.06 | 0.36 | 0.55 | 0.29 | 0.17 | 0.35 | 0.02 | 0.02 | 0.11 | 0.40 | 0.04 | 0.04 | 0.06 | 0.80 | 0.99 | 0.92 | 0.93 | 0.81 | 0.99 | 0.89 | 0.92 |
| AES | 0.34 | 0.48 | 0.20 | 0.46 | 0.02 | 0.01 | 0.02 | 0.02 | 0.02 | 0.00 | 0.00 | 0.01 | 0.01 | 0.00 | 0.00 | 0.00 | 0.67 | 0.00 | 0.81 | 0.92 | 0.65 | 0.00 | 0.72 | 0.05 |
| SZ | 0.27 | 0.88 | 0.67 | 0.96 | 0.00 | 0.11 | 0.00 | 0.02 | 0.01 | 0.00 | 0.00 | 0.00 | 0.00 | 0.00 | 0.00 | 0.00 | 0.00 | 0.08 | 0.00 | 0.56 | 0.00 | 0.23 | 0.00 | 0.43 |
| HLG | 0.37 | 0.62 | 0.36 | 0.63 | 0.33 | 0.56 | 0.46 | 1.00 | 0.25 | 0.09 | 0.07 | 0.33 | 0.29 | 1.00 | 0.55 | 0.99 | 0.94 | 0.97 | 0.95 | 1.00 | 0.95 | 0.87 | 0.92 | 1.00 |
| KL | 0.92 | 0.42 | 0.96 | 0.22 | 0.09 | 0.46 | 0.07 | 0.22 | 0.11 | 0.00 | 0.00 | 0.03 | 0.21 | 0.02 | 0.01 | 0.02 | 0.69 | 0.98 | 0.69 | 0.34 | 0.70 | 0.95 | 0.71 | 0.55 |
| DL | 0.20 | 0.35 | 0.56 | 0.18 | 0.06 | 0.38 | 0.06 | 0.11 | 0.11 | 0.00 | 0.00 | 0.03 | 0.12 | 0.03 | 0.01 | 0.03 | 0.73 | 0.92 | 0.74 | 0.33 | 0.74 | 0.91 | 0.73 | 0.88 |
| ZLU | 0.66 | 0.68 | 0.78 | 0.58 | 0.04 | 0.33 | 0.04 | 0.12 | 0.10 | 0.00 | 0.00 | 0.02 | 0.10 | 0.02 | 0.01 | 0.02 | 0.57 | 0.90 | 0.72 | 0.54 | 0.51 | 0.87 | 0.77 | 0.71 |
| HS | 0.91 | 0.51 | 0.97 | 0.36 | 0.46 | 0.21 | 0.50 | 0.22 | 0.50 | 0.11 | 0.16 | 0.61 | 0.76 | 0.72 | 0.91 | 1.00 | 0.90 | 0.97 | 0.97 | 0.97 | 0.94 | 0.99 | 0.95 | 0.95 |
| LX | 0.53 | 0.42 | 0.60 | 0.57 | 0.15 | 0.40 | 0.11 | 0.20 | 0.19 | 0.01 | 0.01 | 0.06 | 0.17 | 0.02 | 0.02 | 0.03 | 0.67 | 0.97 | 0.90 | 0.68 | 0.66 | 0.97 | 0.85 | 0.69 |
| XLH | 0.22 | 0.79 | 0.59 | 0.66 | 0.03 | 0.18 | 0.05 | 0.04 | 0.03 | 0.02 | 0.00 | 0.04 | 0.03 | 0.03 | 0.02 | 0.03 | 0.49 | 0.76 | 0.87 | 0.85 | 0.51 | 0.69 | 0.81 | 0.81 |
| XBY | 0.00 | 1.00 | 0.42 | 0.84 | 0.00 | 0.17 | 0.00 | 0.04 | 0.02 | 0.00 | 0.00 | 0.00 | 0.00 | 0.01 | 0.00 | 0.00 | 0.38 | 0.41 | 0.43 | 0.61 | 0.41 | 0.38 | 0.65 | 0.23 |
| SY | 0.43 | 0.73 | 0.85 | 0.78 | 0.01 | 0.13 | 0.01 | 0.03 | 0.01 | 0.00 | 0.00 | 0.00 | 0.00 | 0.00 | 0.00 | 0.00 | 0.35 | 0.53 | 0.57 | 0.80 | 0.35 | 0.46 | 0.50 | 0.68 |
| XH | 0.30 | 0.65 | 0.78 | 0.53 | 0.01 | 0.36 | 0.01 | 0.04 | 0.03 | 0.00 | 0.00 | 0.00 | 0.03 | 0.02 | 0.00 | 0.02 | 0.65 | 0.67 | 0.60 | 0.63 | 0.65 | 0.56 | 0.59 | 0.80 |
| CBE | 0.19 | 0.47 | 0.27 | 0.61 | 0.01 | 0.14 | 0.01 | 0.05 | 0.02 | 0.00 | 0.00 | 0.01 | 0.02 | 0.01 | 0.00 | 0.01 | 0.56 | 0.67 | 0.81 | 0.79 | 0.48 | 0.61 | 0.74 | 0.62 |
| XW | 0.24 | 0.62 | 0.51 | 0.65 | 0.00 | 0.30 | 0.01 | 0.05 | 0.04 | 0.00 | 0.00 | 0.01 | 0.02 | 0.01 | 0.00 | 0.01 | 0.41 | 0.70 | 0.53 | 0.83 | 0.41 | 0.74 | 0.67 | 0.81 |
| AR | 0.21 | 0.19 | 0.48 | 0.28 | 0.08 | 0.08 | 0.07 | 0.15 | 0.16 | 0.00 | 0.00 | 0.02 | 0.25 | 0.01 | 0.01 | 0.02 | 0.53 | 0.85 | 0.67 | 0.85 | 0.68 | 0.88 | 0.65 | 0.30 |
| KAQ | 0.90 | 0.40 | 0.87 | 0.16 | 0.30 | 0.34 | 0.22 | 0.16 | 0.14 | 0.01 | 0.01 | 0.06 | 0.22 | 0.06 | 0.03 | 0.06 | 0.75 | 0.98 | 0.93 | 0.94 | 0.83 | 1.00 | 0.92 | 0.90 |
| BLZ | 0.56 | 0.46 | 0.67 | 0.66 | 0.12 | 0.39 | 0.11 | 0.17 | 0.12 | 0.01 | 0.01 | 0.03 | 0.17 | 0.02 | 0.02 | 0.03 | 0.55 | 0.99 | 0.58 | 0.56 | 0.55 | 0.98 | 0.82 | 0.80 |
| ZL | 0.20 | 0.44 | 0.57 | 0.31 | 0.02 | 0.30 | 0.02 | 0.06 | 0.04 | 0.00 | 0.00 | 0.01 | 0.03 | 0.01 | 0.00 | 0.01 | 0.42 | 0.57 | 0.78 | 0.47 | 0.42 | 0.62 | 0.83 | 0.80 |
| AH | 0.91 | 0.57 | 0.98 | 0.35 | 0.16 | 0.32 | 0.13 | 0.19 | 0.19 | 0.01 | 0.01 | 0.04 | 0.26 | 0.02 | 0.02 | 0.03 | 0.66 | 0.99 | 0.89 | 0.77 | 0.59 | 0.98 | 0.87 | 0.67 |
| MZL | 0.07 | 0.95 | 0.33 | 0.79 | 0.60 | 0.40 | 1.00 | 0.07 | 0.43 | 0.11 | 0.25 | 0.79 | 0.45 | 0.29 | 1.00 | 0.73 | 0.85 | 0.83 | 1.00 | 0.94 | 1.00 | 0.96 | 1.00 | 0.97 |
| SS | 0.83 | 0.54 | 0.87 | 0.40 | 0.39 | 0.37 | 0.27 | 0.16 | 0.21 | 0.01 | 0.01 | 0.06 | 0.38 | 0.06 | 0.04 | 0.07 | 0.57 | 0.99 | 0.89 | 0.89 | 0.69 | 0.97 | 0.95 | 0.79 |
| KLU | 0.81 | 0.72 | 0.99 | 0.62 | 0.19 | 0.42 | 0.17 | 0.40 | 0.55 | 0.01 | 0.01 | 0.11 | 0.63 | 0.06 | 0.04 | 0.08 | 0.64 | 0.94 | 0.78 | 0.25 | 0.64 | 0.91 | 0.69 | 0.75 |
| GH | 0.28 | 0.33 | 0.00 | 0.32 | 0.02 | 0.00 | 0.01 | 0.00 | 0.01 | 0.00 | 0.00 | 0.01 | 0.02 | 0.00 | 0.00 | 0.00 | 0.65 | 0.12 | 0.71 | 0.93 | 0.72 | 0.14 | 0.71 | 0.74 |
| BLY | 0.68 | 0.60 | 0.80 | 0.68 | 0.05 | 0.32 | 0.04 | 0.11 | 0.05 | 0.00 | 0.00 | 0.01 | 0.04 | 0.01 | 0.01 | 0.01 | 0.51 | 0.98 | 0.78 | 0.58 | 0.51 | 0.94 | 0.70 | 0.73 |
| XBZ | 0.00 | 0.68 | 0.35 | 0.74 | 0.00 | 0.17 | 0.00 | 0.05 | 0.02 | 0.00 | 0.00 | 0.00 | 0.01 | 0.00 | 0.00 | 0.00 | 0.58 | 0.56 | 0.69 | 0.74 | 0.46 | 0.49 | 0.64 | 0.59 |
| EL | 0.44 | 0.95 | 0.85 | 1.00 | 0.00 | 0.27 | 0.04 | 0.01 | 0.00 | 0.00 | 0.01 | 0.02 | 0.00 | 0.02 | 0.04 | 0.03 | 0.45 | 0.45 | 0.70 | 0.88 | 0.46 | 0.43 | 0.75 | 0.77 |
| JLD | 0.43 | 0.64 | 0.65 | 0.50 | 0.09 | 0.28 | 0.08 | 0.11 | 0.15 | 0.00 | 0.00 | 0.03 | 0.18 | 0.00 | 0.01 | 0.01 | 0.59 | 0.96 | 0.73 | 0.75 | 0.60 | 0.92 | 0.65 | 0.64 |
| NM | 0.89 | 0.63 | 1.00 | 0.40 | 0.12 | 0.34 | 0.10 | 0.20 | 0.18 | 0.01 | 0.01 | 0.05 | 0.19 | 0.02 | 0.02 | 0.03 | 0.73 | 0.99 | 0.82 | 0.48 | 0.73 | 0.97 | 0.88 | 0.79 |
| HLE | 0.26 | 0.31 | 0.25 | 0.60 | 0.45 | 0.31 | 0.58 | 0.19 | 0.30 | 0.07 | 0.11 | 0.41 | 0.37 | 0.24 | 0.35 | 0.36 | 0.93 | 0.89 | 0.95 | 0.93 | 0.92 | 0.93 | 0.95 | 0.94 |
| EWK | 0.22 | 0.44 | 0.27 | 0.59 | 0.02 | 0.11 | 0.03 | 0.04 | 0.03 | 0.00 | 0.00 | 0.01 | 0.02 | 0.01 | 0.00 | 0.01 | 0.27 | 0.62 | 0.76 | 0.78 | 0.27 | 0.71 | 0.67 | 0.43 |
| DW | 0.15 | 0.73 | 0.43 | 0.74 | 0.00 | 0.22 | 0.00 | 0.03 | 0.02 | 0.00 | 0.00 | 0.00 | 0.01 | 0.01 | 0.00 | 0.00 | 0.17 | 0.46 | 0.48 | 0.62 | 0.17 | 0.47 | 0.32 | 0.73 |
| MLD | 0.03 | 0.19 | 0.47 | 0.10 | 0.07 | 0.06 | 0.06 | 0.14 | 0.13 | 0.00 | 0.00 | 0.02 | 0.25 | 0.01 | 0.01 | 0.02 | 0.62 | 0.92 | 0.77 | 0.87 | 0.80 | 0.92 | 0.82 | 0.00 |
| WL | 0.55 | 0.62 | 0.74 | 0.53 | 0.17 | 0.16 | 0.18 | 0.13 | 0.17 | 0.04 | 0.04 | 0.19 | 0.20 | 0.07 | 0.10 | 0.11 | 0.68 | 0.86 | 0.86 | 0.89 | 0.70 | 0.88 | 0.82 | 0.81 |
| ZLT | 0.34 | 0.42 | 0.48 | 0.38 | 0.08 | 0.09 | 0.07 | 0.08 | 0.08 | 0.00 | 0.00 | 0.02 | 0.14 | 0.01 | 0.01 | 0.02 | 0.78 | 0.82 | 0.75 | 0.85 | 0.46 | 0.78 | 0.67 | 0.56 |
| ZXB | 0.18 | 0.52 | 0.69 | 0.47 | 0.02 | 0.35 | 0.02 | 0.05 | 0.03 | 0.00 | 0.00 | 0.01 | 0.04 | 0.01 | 0.00 | 0.01 | 0.46 | 0.64 | 0.56 | 0.43 | 0.47 | 0.66 | 0.76 | 0.52 |
| WNT | 0.81 | 0.63 | 0.94 | 0.55 | 0.09 | 0.27 | 0.08 | 0.14 | 0.13 | 0.00 | 0.00 | 0.03 | 0.18 | 0.01 | 0.01 | 0.02 | 0.55 | 0.96 | 0.79 | 0.72 | 0.52 | 0.94 | 0.74 | 0.73 |
| KZZ | 0.75 | 0.54 | 0.91 | 0.44 | 0.09 | 0.43 | 0.08 | 0.15 | 0.17 | 0.00 | 0.01 | 0.03 | 0.23 | 0.02 | 0.02 | 0.03 | 0.69 | 0.96 | 0.81 | 0.17 | 0.68 | 0.93 | 0.86 | 0.63 |
| ELC | 0.11 | 0.00 | 0.31 | 0.13 | 0.02 | 0.00 | 0.03 | 0.01 | 0.01 | 0.00 | 0.00 | 0.00 | 0.01 | 0.00 | 0.00 | 0.00 | 0.39 | 0.32 | 0.37 | 0.67 | 0.40 | 0.11 | 0.55 | 0.56 |
| ZLN | 0.06 | 0.91 | 0.34 | 0.81 | 1.00 | 0.18 | 0.97 | 0.05 | 0.80 | 1.00 | 1.00 | 1.00 | 0.47 | 0.33 | 0.56 | 0.45 | 1.00 | 0.67 | 0.96 | 0.99 | 0.98 | 0.91 | 0.95 | 0.97 |
| TP | 0.11 | 0.41 | 0.60 | 0.20 | 0.11 | 0.31 | 0.07 | 0.08 | 0.17 | 0.01 | 0.01 | 0.05 | 0.19 | 0.01 | 0.01 | 0.02 | 0.80 | 0.99 | 0.90 | 0.24 | 0.80 | 0.97 | 0.88 | 0.77 |

**Table S7.** Input data for the Hasse diagram (2000-2010).

| League | County | Natural | Human activities | Economic | Urbanisation | Dominant driver | League | County | Natural | Human activities | Economic | Urbanisation | Dominant driver |
| --- | --- | --- | --- | --- | --- | --- | --- | --- | --- | --- | --- | --- | --- |
| Hulunbuir | YKS | 7 | 7 | 8 | 2 | Urbanisation | Hinggan | KYZ | 3 | 5 | 9 | 3 | Natural & Urbanisation |
|  | ERG | 4 | 11 | 13 | 3 | Urbanisation |  | TQ | 4 | 3 | 3 | 2 | Urbanisation |
|  | XBY | 1 | 11 | 14 | 4 | Natural |  | KYQ | 5 | 7 | 8 | 3 | Urbanisation |
|  | CBE | 4 | 9 | 12 | 3 | Urbanisation |  | AES | 6 | 10 | 13 | 2 | Urbanisation |
|  | AR | 7 | 5 | 5 | 3 | Urbanisation |  | JLD | 4 | 5 | 6 | 2 | Urbanisation |
|  | MZL | 2 | 1 | 1 | 1 | Human activities & Economic & Urbanisation |  | WL | 3 | 4 | 3 | 2 | Urbanisation |
|  | GH | 8 | 11 | 13 | 2 | Urbanisation | Tongliao | KEQ | 2 | 1 | 1 | 2 | Human activities & Economic |
|  | XBZ | 4 | 10 | 14 | 3 | Urbanisation |  | HLG | 3 | 1 | 1 | 1 | Human activities & Economic & Urbanisation |
|  | HLE | 4 | 1 | 2 | 1 | Human activities & Urbanisation |  | KL | 1 | 2 | 6 | 3 | Natural |
|  | EWK | 4 | 9 | 11 | 3 | Urbanisation |  | KZH | 3 | 3 | 5 | 3 | Natural & Human activities & Urbanisation |
|  | MLD | 8 | 6 | 6 | 2 | Urbanisation |  | ZLU | 2 | 6 | 7 | 2 | Natural & Urbanisation |
|  | ZLT | 6 | 6 | 7 | 2 | Urbanisation |  | NM | 1 | 4 | 4 | 2 | Natural |
|  | ELC | 8 | 10 | 15 | 4 | Urbanisation |  | KZZ | 3 | 3 | 5 | 3 | Natural & Human activities & Urbanisation |
|  | ZLN | 2 | 1 | 1 | 1 | Human activities & Economic & Urbanisation |  | KLU | 1 | 2 | 2 | 3 | Natural |
| Chifeng | AL | 1 | 4 | 8 | 3 | Natural | Xilingol | AB | 3 | 11 | 14 | 4 | Natural |
|  | KSK | 5 | 8 | 9 | 3 | Urbanisation |  | SZ | 2 | 12 | 16 | 5 | Natural |
|  | YB | 1 | 1 | 1 | 1 | Natural & Human activities & Economic & Urbanisation |  | DL | 6 | 5 | 5 | 2 | Urbanisation |
|  | NC | ISO | 2 | 2 | 1 | Urbanisation |  | XLH | 3 | 6 | 4 | 2 | Urbanisation |
|  | HS | 2 | 2 | 1 | 1 | Economic & Urbanisation |  | SY | 2 | 10 | 16 | 3 | Natural |
|  | LX | 4 | 3 | 4 | 2 | Urbanisation |  | XH | 3 | 6 | 8 | 3 | Natural & Urbanisation |
|  | KAQ | 3 | 3 | 4 | 1 | Urbanisation |  | XW | 3 | 9 | 10 | 3 | Natural & Urbanisation |
|  | BLZ | 3 | 4 | 5 | 2 | Urbanisation |  | ZL | 5 | 8 | 9 | 2 | Urbanisation |
|  | AH | 1 | 3 | 4 | 2 | Natural |  | EL | 1 | 8 | 4 | 3 | Natural |
|  | SS | 2 | 2 | 3 | 1 | Urbanisation |  | DW | 3 | 10 | 13 | 4 | Natural |
|  | BLY | 2 | 6 | 8 | 2 | Natural & Urbanisation |  | ZXB | 5 | 6 | 9 | 4 | Urbanisation |
|  | WNT | 2 | 5 | 6 | 2 | Natural & Urbanisation |  | TP | 6 | 5 | 5 | 2 | Urbanisation |

**Table S8.** Rankings and dominant drivers of LD in EIM for the period 2000–2010.

| County | TM_2010 | PRE_2010 | TM_2020 | PRE_2020 | PN_2010 | Sh_2010 | PN_2020 | Sh_2017 | Dy_2010 | De_2010 | Ds_2010 | GDP_2010 | Dy_2020 | De_2020 | Ds_2020 | GDP_2020 | Cs_2010 | Nc_2010 | Dl_2010 | Kq_2010 | Cs_2020 | Nc_2020 | Dl_2020 | Kq_2020 |
| --- | --- | --- | --- | --- | --- | --- | --- | --- | --- | --- | --- | --- | --- | --- | --- | --- | --- | --- | --- | --- | --- | --- | --- | --- |
| AL | 0.89 | 0.70 | 0.90 | 0.62 | 0.04 | 0.12 | 0.02 | 0.42 | 0.07 | 0.01 | 0.01 | 0.01 | 0.04 | 0.01 | 0.01 | 0.01 | 0.51 | 0.93 | 0.71 | 0.78 | 0.51 | 0.92 | 0.72 | 0.63 |
| KYZ | 0.71 | 0.56 | 0.83 | 0.52 | 0.04 | 0.13 | 0.02 | 0.45 | 0.07 | 0.00 | 0.00 | 0.01 | 0.06 | 0.00 | 0.00 | 0.01 | 0.64 | 0.91 | 0.83 | 0.65 | 0.54 | 0.92 | 0.83 | 0.69 |
| TQ | 0.72 | 0.55 | 0.86 | 0.46 | 0.11 | 0.13 | 0.05 | 0.52 | 0.26 | 0.01 | 0.01 | 0.02 | 0.20 | 0.02 | 0.01 | 0.02 | 0.74 | 0.95 | 0.83 | 0.53 | 0.62 | 0.95 | 0.69 | 0.63 |
| KSK | 0.50 | 0.35 | 0.62 | 0.50 | 0.02 | 0.07 | 0.01 | 0.16 | 0.04 | 0.01 | 0.00 | 0.01 | 0.03 | 0.01 | 0.00 | 0.01 | 0.41 | 0.80 | 0.78 | 0.76 | 0.41 | 0.81 | 0.77 | 0.44 |
| KEQ | 0.96 | 0.47 | 0.99 | 0.42 | 0.40 | 0.44 | 0.18 | 0.64 | 0.94 | 0.36 | 0.24 | 0.41 | 0.36 | 0.16 | 0.21 | 0.21 | 0.74 | 0.97 | 0.94 | 0.89 | 0.76 | 0.98 | 0.97 | 0.80 |
| YKS | 0.13 | 0.36 | 0.16 | 0.40 | 0.03 | 0.01 | 0.01 | 0.04 | 0.07 | 0.01 | 0.01 | 0.01 | 0.02 | 0.00 | 0.01 | 0.00 | 0.74 | 0.68 | 0.82 | 0.58 | 0.75 | 0.46 | 0.87 | 0.24 |
| KYQ | 0.54 | 0.49 | 0.71 | 0.21 | 0.07 | 0.11 | 0.02 | 0.49 | 0.10 | 0.00 | 0.00 | 0.01 | 0.08 | 0.00 | 0.00 | 0.01 | 0.65 | 0.87 | 0.77 | 0.59 | 0.53 | 0.90 | 0.89 | 0.60 |
| YB | 0.98 | 0.39 | 0.94 | 0.54 | 0.54 | 0.39 | 0.33 | 0.33 | 1.00 | 0.38 | 0.29 | 0.45 | 0.66 | 0.38 | 0.19 | 0.29 | 0.89 | 0.99 | 0.96 | 0.98 | 0.87 | 0.99 | 0.99 | 0.96 |
| AB | 0.57 | 0.83 | 0.44 | 0.85 | 0.00 | 0.03 | 0.00 | 0.13 | 0.01 | 0.00 | 0.00 | 0.00 | 0.00 | 0.00 | 0.00 | 0.00 | 0.11 | 0.43 | 0.01 | 0.67 | 0.10 | 0.44 | 0.27 | 0.33 |
| KZH | 0.93 | 0.00 | 0.97 | 0.47 | 0.08 | 0.17 | 0.03 | 0.21 | 0.15 | 0.01 | 0.01 | 0.02 | 0.11 | 0.01 | 0.01 | 0.02 | 0.43 | 0.96 | 0.79 | 0.74 | 0.60 | 0.96 | 0.83 | 0.50 |
| ERG | 0.14 | 0.57 | 0.10 | 0.66 | 0.01 | 0.01 | 0.00 | 0.06 | 0.02 | 0.00 | 0.00 | 0.00 | 0.01 | 0.00 | 0.00 | 0.00 | 0.65 | 0.55 | 0.67 | 0.69 | 0.72 | 0.46 | 0.68 | 0.67 |
| NC | 0.91 | 0.06 | 0.96 | 0.57 | 0.25 | 0.17 | 0.13 | 0.26 | 0.40 | 0.04 | 0.04 | 0.06 | 0.29 | 0.04 | 0.05 | 0.06 | 0.81 | 1.00 | 0.90 | 0.92 | 0.81 | 0.97 | 0.94 | 0.81 |
| AES | 0.18 | 0.44 | 0.35 | 0.04 | 0.03 | 0.02 | 0.01 | 0.07 | 0.01 | 0.00 | 0.00 | 0.00 | 0.01 | 0.00 | 0.00 | 0.00 | 0.54 | 0.00 | 0.68 | 0.00 | 0.55 | 0.22 | 0.74 | 0.52 |
| SZ | 0.66 | 0.95 | 0.55 | 0.95 | 0.00 | 0.02 | 0.00 | 0.06 | 0.00 | 0.00 | 0.00 | 0.00 | 0.00 | 0.00 | 0.00 | 0.00 | 0.00 | 0.11 | 0.00 | 0.47 | 0.00 | 0.00 | 0.00 | 0.00 |
| HLG | 0.34 | 0.64 | 0.43 | 0.53 | 0.32 | 1.00 | 0.27 | 1.00 | 0.29 | 1.00 | 0.55 | 0.99 | 1.00 | 0.03 | 0.14 | 0.15 | 0.94 | 0.84 | 0.89 | 1.00 | 0.94 | 0.93 | 0.98 | 1.00 |
| KL | 0.96 | 0.24 | 1.00 | 0.63 | 0.07 | 0.22 | 0.03 | 0.32 | 0.21 | 0.02 | 0.01 | 0.02 | 0.01 | 0.00 | 0.02 | 0.05 | 0.70 | 0.94 | 0.70 | 0.58 | 0.69 | 0.97 | 0.75 | 0.51 |
| DL | 0.56 | 0.20 | 0.50 | 0.65 | 0.05 | 0.11 | 0.03 | 0.05 | 0.12 | 0.03 | 0.01 | 0.03 | 0.07 | 0.02 | 0.01 | 0.02 | 0.77 | 0.92 | 0.76 | 0.90 | 0.76 | 0.95 | 0.80 | 0.81 |
| ZLU | 0.78 | 0.60 | 0.88 | 0.58 | 0.04 | 0.12 | 0.02 | 0.44 | 0.10 | 0.02 | 0.01 | 0.02 | 0.06 | 0.01 | 0.01 | 0.01 | 0.55 | 0.90 | 0.80 | 0.69 | 0.60 | 0.93 | 0.79 | 0.69 |
| HS | 0.98 | 0.39 | 0.96 | 0.50 | 1.00 | 0.22 | 1.00 | 0.26 | 0.76 | 0.72 | 0.91 | 1.00 | 0.48 | 1.00 | 1.00 | 1.00 | 0.97 | 0.99 | 0.97 | 0.95 | 0.95 | 1.00 | 0.99 | 0.92 |
| LX | 0.63 | 0.59 | 0.68 | 0.52 | 0.11 | 0.20 | 0.06 | 0.45 | 0.17 | 0.02 | 0.02 | 0.03 | 0.12 | 0.04 | 0.03 | 0.04 | 0.71 | 0.96 | 0.86 | 0.69 | 0.71 | 0.95 | 0.97 | 0.45 |
| XLH | 0.59 | 0.71 | 0.49 | 0.69 | 0.04 | 0.04 | 0.03 | 0.16 | 0.03 | 0.03 | 0.02 | 0.03 | 0.03 | 0.03 | 0.02 | 0.03 | 0.44 | 0.67 | 0.76 | 0.81 | 0.49 | 0.69 | 0.90 | 0.71 |
| XBY | 0.40 | 0.86 | 0.34 | 0.77 | 0.00 | 0.04 | 0.00 | 0.19 | 0.00 | 0.01 | 0.00 | 0.00 | 0.01 | 0.01 | 0.00 | 0.00 | 0.48 | 0.32 | 0.66 | 0.28 | 0.47 | 0.36 | 0.43 | 0.46 |
| SY | 0.84 | 0.78 | 0.68 | 0.98 | 0.01 | 0.03 | 0.00 | 0.11 | 0.00 | 0.00 | 0.00 | 0.00 | 0.00 | 0.00 | 0.00 | 0.00 | 0.37 | 0.40 | 0.57 | 0.69 | 0.37 | 0.40 | 0.68 | 0.51 |
| XH | 0.77 | 0.52 | 0.60 | 0.90 | 0.01 | 0.04 | 0.00 | 0.15 | 0.03 | 0.02 | 0.00 | 0.02 | 0.02 | 0.01 | 0.00 | 0.01 | 0.63 | 0.51 | 0.58 | 0.81 | 0.63 | 0.56 | 0.54 | 0.65 |
| CBE | 0.27 | 0.66 | 0.19 | 0.59 | 0.02 | 0.05 | 0.00 | 0.16 | 0.02 | 0.01 | 0.00 | 0.01 | 0.02 | 0.01 | 0.00 | 0.01 | 0.55 | 0.59 | 0.78 | 0.68 | 0.62 | 0.63 | 0.83 | 0.47 |
| XW | 0.48 | 0.66 | 0.51 | 0.64 | 0.01 | 0.05 | 0.00 | 0.12 | 0.02 | 0.01 | 0.00 | 0.01 | 0.02 | 0.02 | 0.00 | 0.01 | 0.43 | 0.71 | 0.68 | 0.85 | 0.46 | 0.68 | 0.86 | 0.75 |
| AR | 0.45 | 0.29 | 0.51 | 0.14 | 0.06 | 0.15 | 0.03 | 0.54 | 0.25 | 0.01 | 0.01 | 0.02 | 0.11 | 0.00 | 0.01 | 0.01 | 0.67 | 0.85 | 0.62 | 0.33 | 0.53 | 0.77 | 0.75 | 0.25 |
| KAQ | 0.82 | 0.16 | 0.94 | 0.52 | 0.19 | 0.16 | 0.10 | 0.30 | 0.22 | 0.06 | 0.03 | 0.06 | 0.15 | 0.05 | 0.04 | 0.05 | 0.80 | 0.99 | 0.92 | 0.87 | 0.78 | 0.98 | 0.93 | 0.73 |
| BLZ | 0.71 | 0.70 | 0.82 | 0.66 | 0.11 | 0.17 | 0.05 | 0.63 | 0.17 | 0.02 | 0.02 | 0.03 | 0.12 | 0.03 | 0.02 | 0.03 | 0.67 | 0.97 | 0.84 | 0.82 | 0.67 | 0.97 | 0.92 | 0.55 |
| ZL | 0.56 | 0.33 | 0.50 | 0.67 | 0.02 | 0.06 | 0.01 | 0.07 | 0.03 | 0.01 | 0.00 | 0.01 | 0.02 | 0.01 | 0.00 | 0.01 | 0.44 | 0.59 | 0.79 | 0.80 | 0.44 | 0.63 | 0.85 | 0.56 |
| AH | 0.99 | 0.41 | 0.94 | 0.70 | 0.12 | 0.19 | 0.06 | 0.37 | 0.26 | 0.02 | 0.02 | 0.03 | 0.17 | 0.01 | 0.02 | 0.03 | 0.60 | 0.98 | 0.89 | 0.68 | 0.67 | 0.99 | 0.94 | 0.25 |
| MZL | 0.31 | 0.81 | 0.21 | 0.75 | 0.75 | 0.07 | 0.32 | 0.23 | 0.45 | 0.29 | 1.00 | 0.73 | 0.20 | 0.34 | 0.55 | 0.47 | 1.00 | 0.91 | 1.00 | 0.99 | 0.97 | 0.68 | 1.00 | 0.98 |
| SS | 0.84 | 0.40 | 0.87 | 0.48 | 0.22 | 0.16 | 0.14 | 0.31 | 0.38 | 0.06 | 0.04 | 0.07 | 0.26 | 0.06 | 0.08 | 0.08 | 0.66 | 0.97 | 0.94 | 0.78 | 0.57 | 0.97 | 0.85 | 0.56 |
| KLU | 0.99 | 0.63 | 0.98 | 0.60 | 0.16 | 0.40 | 0.07 | 0.71 | 0.63 | 0.06 | 0.04 | 0.08 | 0.36 | 0.02 | 0.03 | 0.05 | 0.65 | 0.90 | 0.69 | 0.78 | 0.65 | 0.92 | 0.75 | 0.61 |
| GH | 0.00 | 0.32 | 0.00 | 0.50 | 0.01 | 0.00 | 0.00 | 0.00 | 0.02 | 0.00 | 0.00 | 0.00 | 0.00 | 0.00 | 0.00 | 0.00 | 0.66 | 0.33 | 0.65 | 0.70 | 0.56 | 0.24 | 0.63 | 0.68 |
| BLY | 0.82 | 0.70 | 0.83 | 0.59 | 0.04 | 0.11 | 0.02 | 0.65 | 0.04 | 0.01 | 0.01 | 0.01 | 0.03 | 0.01 | 0.01 | 0.01 | 0.52 | 0.94 | 0.74 | 0.73 | 0.51 | 0.98 | 0.88 | 0.57 |
| XBZ | 0.33 | 0.74 | 0.26 | 0.64 | 0.00 | 0.05 | 0.00 | 0.11 | 0.01 | 0.00 | 0.00 | 0.00 | 0.01 | 0.00 | 0.00 | 0.00 | 0.44 | 0.48 | 0.69 | 0.62 | 0.54 | 0.45 | 0.75 | 0.39 |
| EL | 0.85 | 1.00 | 0.66 | 1.00 | 0.06 | 0.01 | 0.03 | 0.04 | 0.00 | 0.02 | 0.04 | 0.03 | 0.00 | 0.01 | 0.04 | 0.03 | 0.52 | 0.52 | 0.64 | 0.78 | 0.64 | 0.51 | 0.67 | 0.54 |
| JLD | 0.63 | 0.51 | 0.73 | 0.22 | 0.07 | 0.11 | 0.03 | 0.41 | 0.18 | 0.00 | 0.01 | 0.01 | 0.14 | 0.00 | 0.01 | 0.01 | 0.60 | 0.92 | 0.66 | 0.65 | 0.68 | 0.95 | 0.70 | 0.42 |
| NM | 1.00 | 0.44 | 1.00 | 0.68 | 0.09 | 0.20 | 0.05 | 0.36 | 0.19 | 0.02 | 0.02 | 0.03 | 0.13 | 0.01 | 0.02 | 0.02 | 0.73 | 0.96 | 0.87 | 0.80 | 0.73 | 0.97 | 0.87 | 0.53 |
| HLE | 0.23 | 0.62 | 0.21 | 0.56 | 0.52 | 0.19 | 0.29 | 0.52 | 0.37 | 0.24 | 0.35 | 0.36 | 0.13 | 0.14 | 0.27 | 0.22 | 0.92 | 0.92 | 0.97 | 0.94 | 0.93 | 0.91 | 0.99 | 0.94 |
| EWK | 0.24 | 0.59 | 0.22 | 0.51 | 0.02 | 0.04 | 0.01 | 0.11 | 0.02 | 0.01 | 0.00 | 0.01 | 0.02 | 0.02 | 0.01 | 0.01 | 0.31 | 0.65 | 0.63 | 0.49 | 0.32 | 0.56 | 0.71 | 0.52 |
| DW | 0.42 | 0.76 | 0.43 | 0.65 | 0.00 | 0.03 | 0.00 | 0.14 | 0.01 | 0.01 | 0.00 | 0.00 | 0.01 | 0.00 | 0.00 | 0.00 | 0.21 | 0.46 | 0.31 | 0.79 | 0.22 | 0.48 | 0.71 | 0.54 |
| MLD | 0.44 | 0.12 | 0.42 | 0.16 | 0.05 | 0.14 | 0.02 | 0.34 | 0.25 | 0.01 | 0.01 | 0.02 | 0.15 | 0.00 | 0.01 | 0.01 | 0.81 | 0.90 | 0.80 | 0.07 | 0.62 | 0.90 | 0.74 | 0.33 |
| WL | 0.73 | 0.54 | 0.85 | 0.17 | 0.13 | 0.13 | 0.14 | 0.49 | 0.20 | 0.07 | 0.10 | 0.11 | 0.17 | 0.13 | 0.10 | 0.12 | 0.64 | 0.79 | 0.74 | 0.82 | 0.67 | 0.81 | 0.77 | 0.78 |
| ZLT | 0.46 | 0.40 | 0.58 | 0.00 | 0.06 | 0.08 | 0.03 | 0.26 | 0.14 | 0.01 | 0.01 | 0.02 | 0.08 | 0.01 | 0.01 | 0.01 | 0.54 | 0.80 | 0.73 | 0.63 | 0.79 | 0.79 | 0.73 | 0.55 |
| ZXB | 0.68 | 0.51 | 0.50 | 0.75 | 0.02 | 0.05 | 0.01 | 0.11 | 0.04 | 0.01 | 0.00 | 0.01 | 0.02 | 0.01 | 0.00 | 0.01 | 0.46 | 0.62 | 0.73 | 0.54 | 0.46 | 0.62 | 0.36 | 0.39 |
| WNT | 0.91 | 0.54 | 0.87 | 0.55 | 0.07 | 0.14 | 0.03 | 0.39 | 0.18 | 0.01 | 0.01 | 0.02 | 0.12 | 0.01 | 0.01 | 0.02 | 0.51 | 0.92 | 0.76 | 0.74 | 0.56 | 0.94 | 0.81 | 0.52 |
| KZZ | 0.90 | 0.41 | 0.93 | 0.51 | 0.08 | 0.15 | 0.04 | 0.42 | 0.23 | 0.02 | 0.02 | 0.03 | 0.17 | 0.01 | 0.01 | 0.02 | 0.71 | 0.94 | 0.85 | 0.64 | 0.72 | 0.95 | 0.88 | 0.61 |
| ELC | 0.30 | 0.14 | 0.28 | 0.37 | 0.04 | 0.01 | 0.01 | 0.02 | 0.01 | 0.00 | 0.00 | 0.00 | 0.01 | 0.00 | 0.00 | 0.00 | 0.48 | 0.22 | 0.58 | 0.57 | 0.46 | 0.35 | 0.44 | 0.10 |
| ZLN | 0.33 | 0.82 | 0.22 | 0.74 | 0.58 | 0.05 | 0.39 | 0.17 | 0.47 | 0.33 | 0.56 | 0.45 | 0.11 | 0.51 | 0.20 | 0.31 | 0.97 | 0.87 | 0.94 | 0.97 | 1.00 | 0.61 | 0.93 | 0.95 |
| TP | 0.58 | 0.22 | 0.46 | 0.67 | 0.06 | 0.08 | 0.03 | 0.11 | 0.19 | 0.01 | 0.01 | 0.02 | 0.12 | 0.01 | 0.02 | 0.02 | 0.78 | 0.99 | 0.87 | 0.80 | 0.78 | 0.99 | 0.82 | 0.76 |

**Table S9.** Input data for the Hasse diagram (2010-2020).

| League | County | Natural | Human activities | Economic | Urbanisation | Dominant driver | League | County | Natural | Human activities | Economic | Urbanisation | Dominant driver |
| --- | --- | --- | --- | --- | --- | --- | --- | --- | --- | --- | --- | --- | --- |
| Hulunbuir | YKS | 7 | 7 | 5 | 3 | Urbanisation | Hinggan | KYZ | 3 | 5 | 6 | 3 | Natural & Urbanisation |
|  | ERG | 5 | 9 | 7 | 3 | Urbanisation |  | TQ | 3 | 4 | 3 | 3 | Natural & Economic & Urbanisation |
|  | XBY | 3 | 7 | 7 | 5 | Natural |  | KYQ | 5 | 5 | 6 | 3 | Urbanisation |
|  | CBE | 6 | 7 | 5 | 4 | Urbanisation |  | AES | 6 | 7 | 7 | 4 | Urbanisation |
|  | AR | 6 | 4 | 4 | 4 | Human activities & Economic & Urbanisation |  | JLD | 4 | 5 | 4 | 4 | Natural & Economic & Urbanisation |
|  | MZL | 4 | 2 | 1 | 1 | Economic & Urbanisation |  | WL | 3 | 2 | 2 | 3 | Human activities & Economic |
|  | GH | 7 | 10 | 7 | 3 | Urbanisation | Tongliao | KEQ | 1 | 1 | 1 | 2 | Natural & Human activities & Economic |
|  | XBZ | 5 | 9 | 7 | 5 | Natural & Urbanisation |  | HLG | 5 | 1 | 1 | 1 | Human activities & Economic & Urbanisation |
|  | HLE | 6 | 1 | 2 | 1 | Human activities & Urbanisation |  | KL | 1 | 3 | 3 | 4 | Natural |
|  | EWK | 6 | 7 | 4 | 5 | Economic |  | KZH | 2 | 5 | 4 | 4 | Natural |
|  | MLD | 6 | 6 | 4 | 2 | Urbanisation |  | ZLU | 2 | 5 | 4 | 3 | Natural |
|  | ZLT | 6 | 6 | 5 | 2 | Urbanisation |  | NM | 1 | 4 | 3 | 3 | Natural |
|  | ELC | 6 | 7 | 7 | 5 | Urbanisation |  | KZZ | 2 | 4 | 3 | 3 | Natural |
|  | ZLN | 4 | 2 | 2 | 1 | Urbanisation |  | KLU | 1 | 2 | 2 | 3 | Natural |
| Chifeng | AL | 1 | 5 | 5 | 3 | Natural | Xilingol | AB | 3 | 9 | 8 | 5 | Natural |
|  | KSK | 4 | 7 | 4 | 4 | Natural & Economic & Urbanisation |  | SZ | 2 | 10 | 9 | 6 | Natural |
|  | YB | 2 | 1 | 1 | 1 | Human activities & Economic & Urbanisation |  | DL | 5 | 5 | 3 | 2 | Urbanisation |
|  | NC | 2 | 2 | 2 | 1 | Urbanisation |  | XLH | 3 | 6 | 3 | 3 | Natural & Economic & Urbanisation |
|  | HS | 2 | 1 | 1 | 1 | Human activities & Economic & Urbanisation |  | SY | 1 | 9 | 7 | 5 | Natural |
|  | LX | 3 | 3 | 3 | 3 | Natural & Human activities & Economic & Urbanisation |  | XH | 2 | 8 | 4 | 4 | Natural |
|  | KAQ | 3 | 3 | 2 | 2 | Economic & Urbanisation |  | XW | 3 | 8 | 4 | 3 | Natural & Urbanisation |
|  | BLZ | 1 | 3 | 3 | 3 | Natural |  | ZL | 4 | 8 | 6 | 3 | Urbanisation |
|  | AH | 1 | 3 | 3 | 2 | Natural |  | EL | 1 | 6 | 3 | 4 | Natural |
|  | SS | 3 | 2 | 2 | 2 | Human activities & Economic & Urbanisation |  | DW | 4 | 9 | 7 | 4 | Natural & Urbanisation |
|  | BLY | 2 | 3 | 6 | 2 | Natural & Urbanisation |  | ZXB | 3 | 8 | 6 | 5 | Natural |
|  | WNT | 2 | 5 | 4 | 4 | Natural |  | TP | 4 | 5 | 4 | 2 | Urbanisation |

**Table S10.** Rankings and dominant drivers of LD in EIM for the period 2010–2020.

**Fig. S**

**Fig. S1.** Temporal changes in the drivers of LD in EIM (1990–2020): (a) annual average temperature; (b) annual total precipitation; (c) total population; (d) number of sheep; (e) GDP; (f) primary, secondary, and tertiary GDP; (g) urban area; (h) rural settlement area; (i) road length; (j) other built-up area.

**Fig. S2.** Research framework. Map and diagram created using ArcGIS 10.8 (Esri, https://www.esri.com) and Visio 2024 (Microsoft, https://www.microsoft.com/).


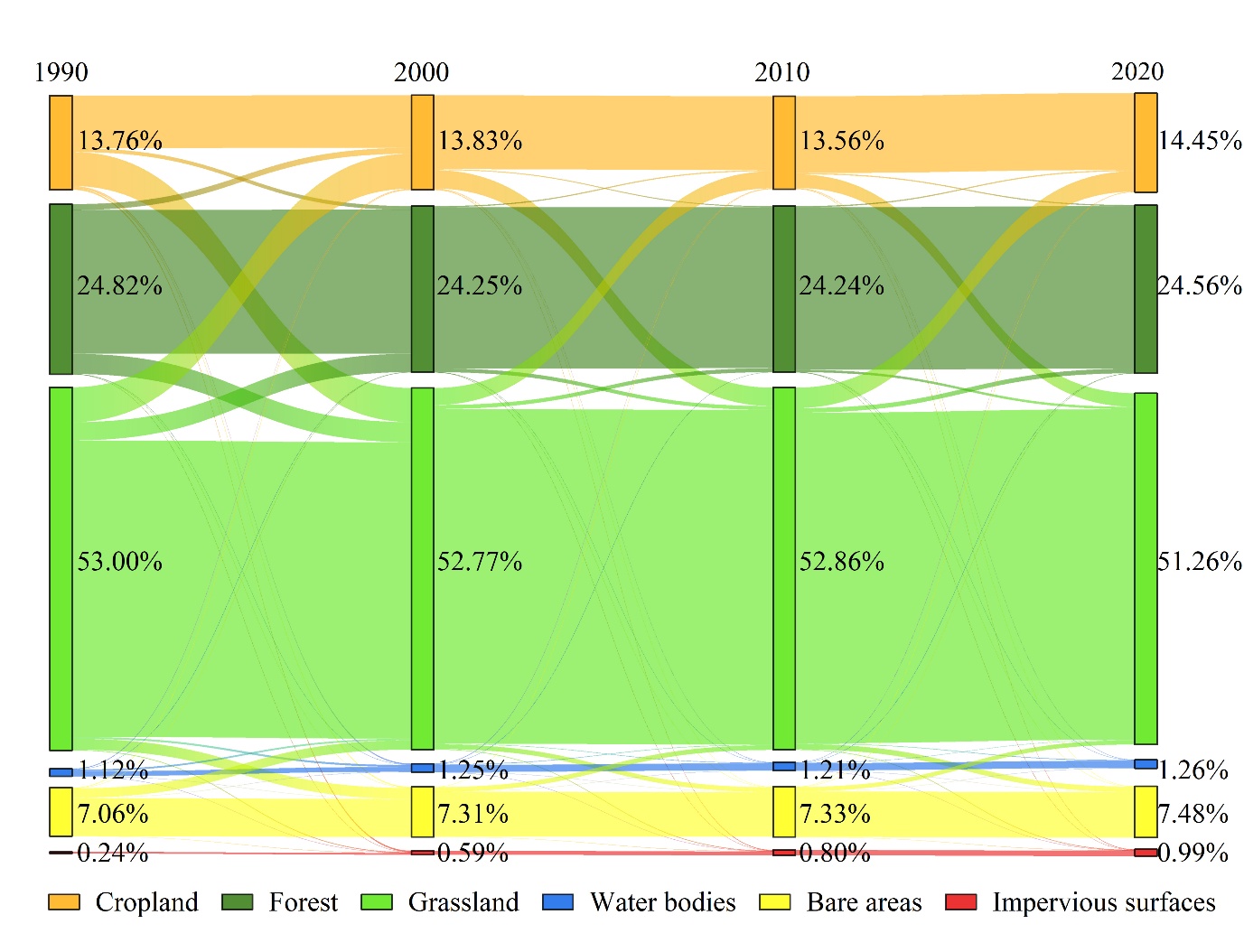


**Fig. S3.** Sankey diagram of land cover type transitions (1990-2020).


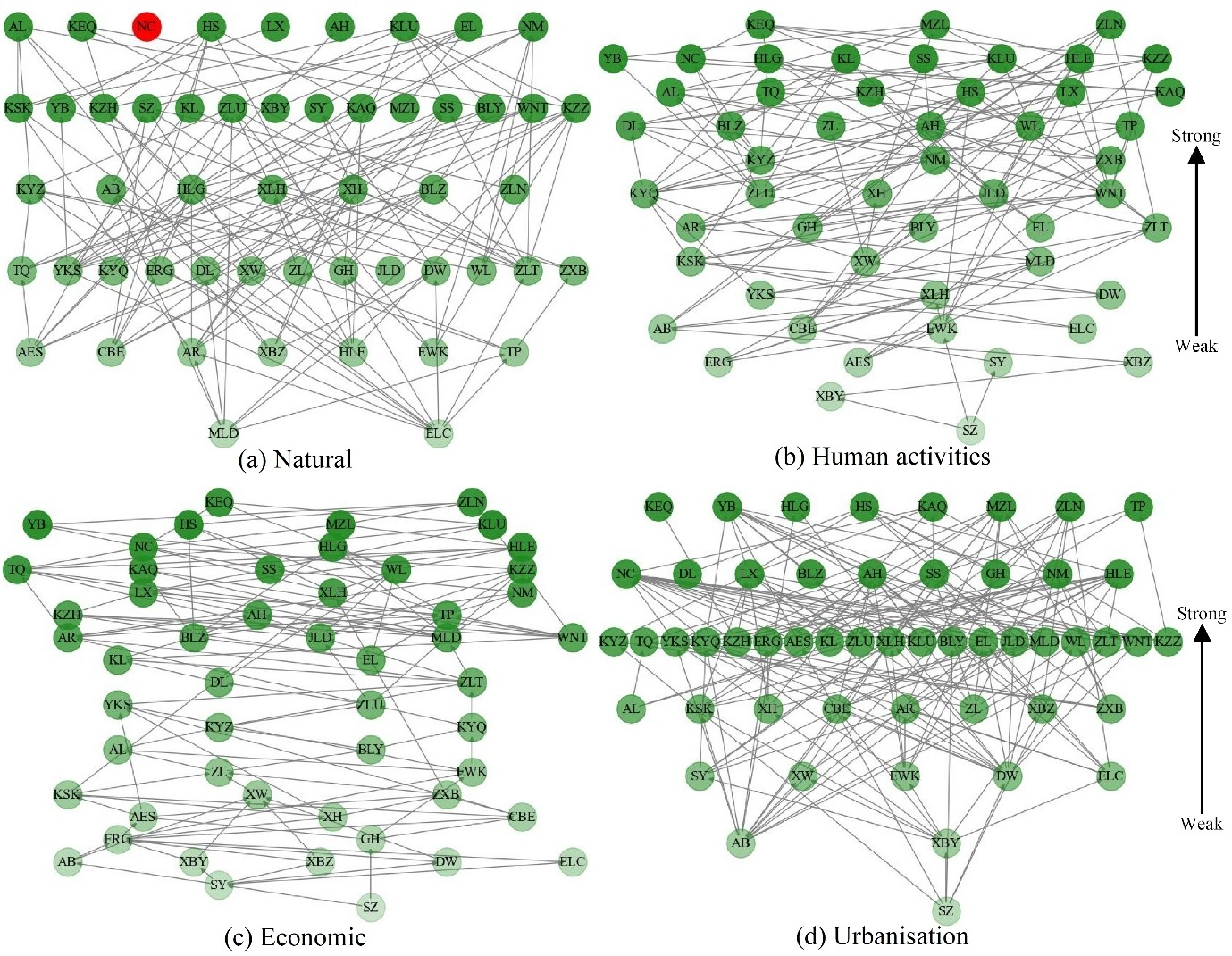


**Fig. S4.** Partial order ranking of LD driving factors in EIM (1990–2000): (a) natural driver group; (b) human activities driver group; (c) economic driver group; (d) urbanisation driver group.


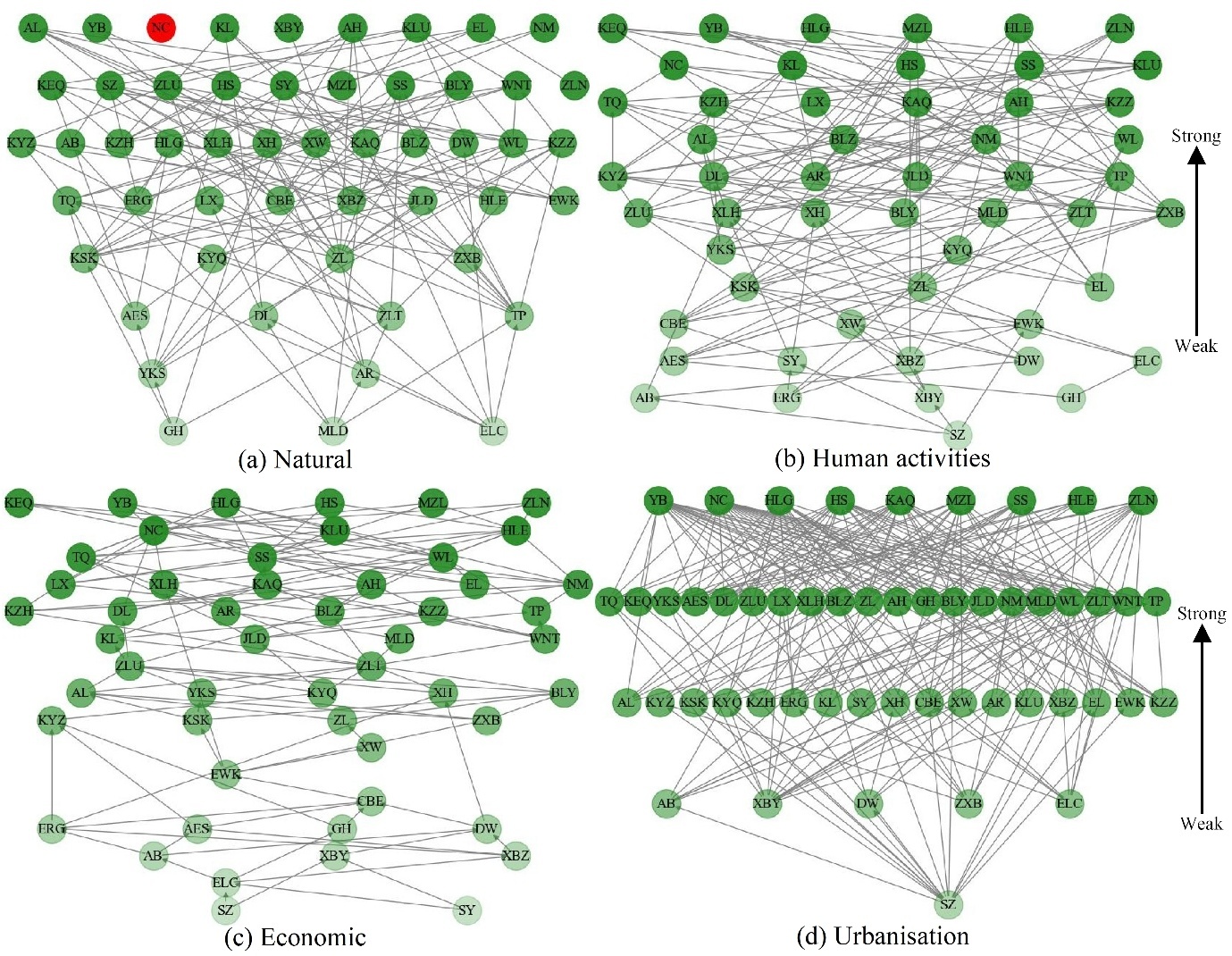


**Fig. S5.** Partial order ranking of LD driving factors in EIM (2000–2010): (a) natural driver group; (b) human activities driver group; (c) economic driver group; (d) urbanisation driver group.


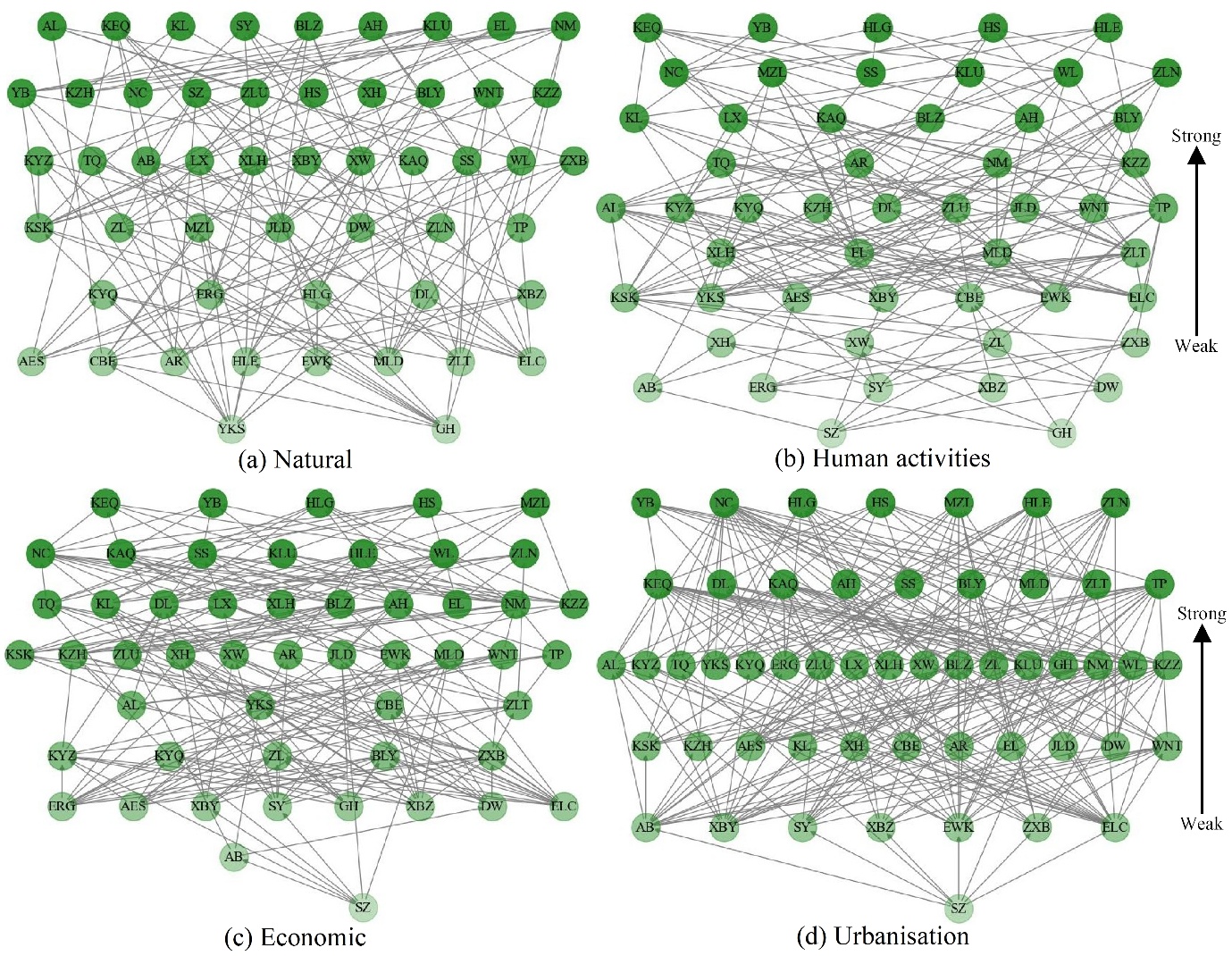


**Fig. S6.** Partial order ranking of LD driving factors in EIM (2010–2020): (a) natural driver group; (b) human activities driver group; (c) economic driver group; (d) urbanisation driver group.
